# Influence of SnWO_4_, SnW_3_O_9_, and WO_3_ Phases in Tin Tungstate Films on Photoelectrochemical Water Oxidation

**DOI:** 10.1021/acsami.4c09713

**Published:** 2024-09-03

**Authors:** Farabi Bozheyev, Steffen Fengler, Jiri Kollmann, Daniel Abou-Ras, Nico Scharnagl, Mauricio Schieda

**Affiliations:** † Institute of Photoelectrochemistry, Helmholtz-Zentrum Hereon GmbH, Max-Planck-Straße 1, 21502 Geesthacht, Germany; ‡ Institute of Applied Science and Information Technologies, Baizakov Street 280, 050042 Almaty, Kazakhstan; § National Nanolaboratory, al-Farabi Kazakh National University, al-Farabi Avenue 71, 050000 Almaty, Kazakhstan; ∥ Department Structure and Dynamics of Energy Materials, Helmholtz-Zentrum Berlin für Materialien und Energie GmbH, Hahn-Meitner-Platz 1, 14109 Berlin, Germany; ⊥ Institute of Surface Science, Helmholtz-Zentrum Hereon GmbH, Max-Planck-Straße 1, 21502 Geesthacht, Germany

**Keywords:** thin films, α-SnWO_4_, SnW_3_O_9_, WO_3_, crystallization, photocurrent density, transient surface photovoltage, photoelectrochemistry

## Abstract

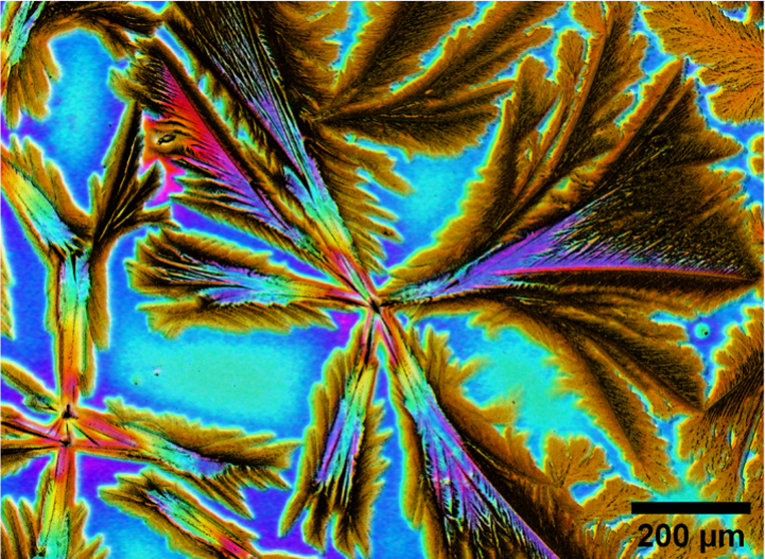

An essential step toward enabling the production of renewable and cost-efficient fuels is an improved understanding of the performance of energy conversion materials. In recent years, there has been growing interest in ternary metal oxides. Particularly, α-SnWO_4_ exhibited promising properties for application to photoelectrochemical (PEC) water splitting. However, the number of corresponding studies remains limited, and a deeper understanding of the physical and chemical processes in α-SnWO_4_ is necessary. To date, charge-carrier generation, separation, and transfer have not been exhaustively studied for SnWO_4_-based photoelectrodes. All of these processes depend on the phase composition, not only α-SnWO_4_ but also on the related phases SnW_3_O_9_ and WO_3_, as well as on their spatial distributions resulting from the coating synthesis. In the present work, these processes in different phases of tin tungstate films were investigated by transient surface photovoltage (TSPV) spectroscopy to complement the analysis of the applicability of α-SnWO_4_ thin films for practical PEC oxygen evolution. Pure α-SnWO_4_ films exhibit higher photoactivities than those of films containing secondary SnW_3_O_9_ and WO_3_ phases due to the higher recombination of charge carriers when these phases are present.

## Introduction

Sustainable hydrogen fuel produced by photoelectrochemical (PEC) water splitting is a promising concept, which could be practically realizable if artificial leaf devices were efficient and stable over time.^1^ In the common PEC configuration, a photoelectrode is in direct contact with the electrolyte that generates hydrogen upon illumination. For instance, a record efficiency of 19% was achieved by a GaInP/GaInAs/GaAs photoelectrode, a system prepared by complex methods and containing expensive and rare elements.^2^ On the other hand, a system based on more abundant elements, BiVO_4_-Fe_2_O_3_-2p c-Si (2p is series-connected two-parallel c-Si solar cells), exhibited an efficiency of only 7.7%.^3^ Another alternative is direct photovoltaic electrolysis that resulted in the highest efficiency of 30% based on complex multijunction GaInP/GaAs/GaInNAsSb/PEM with a light concentrator.^4^ In the current state, still, these approaches are not sufficient for commercialization due to the imbalance between cost, efficiency, and stability.^5^

Over the past 5 years, among the ternary metal oxides, n-type SnWO_4_ was given increased attention owing to its optical properties (*E*_g_ ∼ 1.7–2.0 eV) and its valence and conduction band edge positions suitable for water splitting. Maximum theoretical photocurrent densities of 14.5–22.5 mA cm^–2^, corresponding to solar-to-hydrogen (STH) efficiencies of 25–29% can be expected according to the Shockley–Queisser (S–Q) limit.^6^ However, there are only a limited number of publications including original reports and systematic reviews on α-SnWO_4_ for solar energy applications.^7−25^ In addition, only a handful of methods have been developed to synthesize this material. The main limiting factor for metal oxides is the small polaron hopping mechanism^26^ and ionic point defects that lead to typically low charge-carrier mobilities of 10^–3^–10^–1^ cm^2^ V^–1^ s^–1^. In α-SnWO_4_, furthermore, the carrier diffusion length (*L*_D_ ∼ 10–100 nm) is 10^3^ times smaller than the penetration depth. These limitations may be overcome by nanostructuring and improving the intrinsic crystalline quality, leading to enhanced charge-carrier transport.^23^

Various strategies have been explored in the past to improve the quality, efficiency, and stability of α-SnWO_4_ for PEC water splitting. The orientation of the crystal planes is crucial for efficient water splitting. Naturally, different facets can be expected to have different defect densities and, thus, would also exhibit different properties with respect to nonradiative recombination. Surface reconstructions of different facets also play a role. A chaotic orientation of nanoplatelets on a conductive substrate has been shown to result in an increase of the recombination rate, while an ordered orientation significantly reduced the influence of the recombination on the efficiency.^27^ For instance, a higher proportion of the active facets (100)- vs (001)-planes has been reported to result in a 3-fold photocurrent density increase, up to 0.79 mA cm^–2^ at 1.23 V_RHE_.^28^ Hybrid density functional theory calculations suggest that designing (110)- and (100)-oriented α-SnWO_4_ would increase the photoelectrocatalytic oxygen evolution reaction (OER) and hydrogen evolution reaction (HER).^16^ The formation of oxygen vacancies in a reductive atmosphere has been shown to lead to a significant improvement of the photocurrent.^9^ In addition to improvement in the quality of the bulk, chemical stability improvement is required on the surface. During the testing in an electrolyte, α-SnWO_4_ tends to oxidize, i.e., to form SnO_2_ on the surface, which acts as a hole-blocking layer. Different protection layers such as NiO_*x*_ and CoO_*x*_ were explored as well as operated at different electrolyte pH values.^7,17,19,27^ However, none of these procedures has so far led to a stable performance, due to surface dissolution, showing that there is still a need for suitable protection-coatings.^29^

In the present report, the focus is on understanding the influence of the phase composition of tin tungstate films (α-SnWO_4_, SnW_3_O_9_, and WO_3_) on photocurrent and charge-carrier transfer by means of transient surface photovoltage (TSPV) and photoelectrochemical (PEC) characterization. The main finding is that the photocurrent depends on the composition of the phases in tin tungstate films, which determines the kinetics of charge-carrier generation and recombination.

## Experimental Methods

### Preparation of Films

n-Si, fluorine-doped tin oxide (FTO), and quartz were used as substrates for deposition of SnWO_4_ films. These substrates were cleaned with isopropanol for 10 min in an ultrasound bath, rinsed with distilled water, dried in N_2_ flow, and then exposed to ozone for 10 min. Edges of the substrates were masked using Cu tape before the film deposition for the purpose of film-thickness measurements. For the synthesis of SnWO_4_ films, first, W films were deposited by magnetron sputtering from a W target (99.95%) using Ar gas (Figure S1). The thicknesses of the films were controlled with a quartz crystal microbalance. Then, the W films were annealed at 500 °C in a muffle furnace over a period of 3 h in air to obtain tungsten trioxide (WO_3_) films. Subsequently, the WO_3_ films were placed in an alumina boat within 1–2 cm of 0.5 g of SnCl_2_ powder (also placed in the boat), and the boat was covered with another boat of similar size (Figure S2). The tube was pumped to about 2 × 10^–2^ mbar and then heated to achieve the chemical reaction (Carbolite Gero 30–3000 °C)^10^ at temperatures ranging from 350 to 650 °C during 1 h and cooled afterward. The resulting films were brownish in color, in correspondence with the α-SnWO_4_ phase.

### Characterizations

#### Surface and Structure

The surface morphology of the SnWO_4_ films was studied by an optical microscope (Keyence VK-X260 confocal laser scanning microscope (CLSM)). The structure and phase composition of SnWO_4_ films were investigated by using X-ray diffraction (XRD) in a Bruker D8 Discover X-ray diffractometer. The morphology of SnWO_4_ films was observed by scanning electron microscopy (Zeiss EVO 15). The phonon modes of the SnWO_4_ films were studied by Raman spectroscopy using a WITec alpha300 Raman Microscope with an excitation laser wavelength of 532 nm. Energy dispersive X-ray (EDX) elemental distributions were acquired on polished cross-sectional specimens by using a Zeiss UltraPlus scanning electron microscope equipped with an Oxford Instruments Ultim Extreme EDX detector. The beam energy and current were 7 kV and about 6 nA. The cross-sectional specimens were prepared each by gluing two sample stripes face-to-face together using epoxy glue and then polishing the cross-sectional surface mechanically and by using a focused Ar-ion beam.

#### Optical Spectroscopy and Ellipsometry

Transmittance spectra were obtained by using an Agilent UV–vis Cary5000 spectrometer. Refractive indices (*n*) and extinction coefficients (*k*) were determined by spectroscopic ellipsometry by a J.A. Woollam RC2 ellipsometer. The spectra were measured at three different incident angles of 60, 65, and 70° in a wavelength range between 210 and 2500 nm. Finally, the data were fitted with a Kramers–Kronig consistent basis spline function.

#### TSPV Measurements

Modulated transient surface photovoltage (TSPV) spectra were measured with 8 Hz (125 ms) modulated light in a fixed capacitor arrangement.^30^ The energies for sample excitation were selected by a prism monochromator using the light of a 300 W xenon lamp. An individual TSPV spectrum consists of a series of on-and-off experiments performed at different light excitation energies. The sample was exposed to light and then remained in the dark during the first half and second half of a 125 ms (8 Hz) long measurement period. The FTO-coated quartz electrode and the sample isolated from each other by mica served as the electrodes of the measurement capacitor. The signals of the measurements were recorded by using an oscilloscope card (GaGe 1622 CompuScope).

#### X-ray Photoelectron Spectroscopy (XPS)

XPS measurements were performed using a KRATOS AXIS Ultra DLD (Kratos Analytical, Manchester, U.K.) equipped with a monochromatic Al Kα anode working with 10 mA at 15 kV (150 W). For the survey spectra, a pass energy of 160 eV was used, while for the region spectra, the pass energy was 20 eV. The investigated area was 700 × 300 μm^2^. For depth profiling, Ar etching was performed. The etching rate was about 6 nm min^–1^ related to Ta_2_O_5_ (acceleration voltage 3.8 kV with an extraction current of 100 μA). The evaluation and validation of the data were carried out with the software CASA-XPS version 2.3.25. Calibration of the spectra was done by adjusting the C 1s signal to 284.5 eV. For quantification and deconvolution of the region files, background subtraction (Shirley or U 4 Tougaard) was performed before the calculation.

#### Photoelectrochemical Measurements

Current–voltage (*j*–*V*), chronoamperometry, and open-circuit potential measurements were carried out with an Ametek VersaSTAT 4 Potentiostat. PEC testing was performed in a 3-electrode cell (Zahner PECC2) with a Pt ring counter electrode, Ag/AgCl (3.5 M NaCl, *V*_Ag/AgCl_^0^ = 0.209 V_SHE_) was the reference electrode, and a prepared film on a Si or FTO was the working electrode. The electrolyte was a sodium sulfate (0.5 M Na_2_SO_4_, pH = 7) solution. A strip of Cu tape was fixed on top of the FTO substrates before coating the films to protect the bare contact. Afterward, the Cu tape was removed, and a fresh strip of adhesive Cu tape was connected to this bare contact for the PEC tests. The potential for *j*–*V* measurements was varied from −0.7 to 1.3 V_Ag/AgCl_ with a 25 mV s^–1^ scan rate. A 300 W xenon lamp with an AM1.5G filter was used as the light source. For the chopped *j*–*V* measurements, a shutter between the lamp and the cell was opened and closed for 2 s periods. The voltage V_RHE_, measured against the Ag/AgCl reference electrode, was converted into the reversible hydrogen electrode (RHE) scale using the Nernst equation: *V*_RHE_ = *V*_Ag/AgCl_ + 0.0591 × pH + *V*_Ag/AgCl_^o^, where *V*_Ag/AgCl_ is the applied potential and *V*_Ag/AgCl_^o^ is the standard potential of the Ag/AgCl reference electrode.

## Results and Discussion

### Structural and Morphological Studies

**Figure 1:**
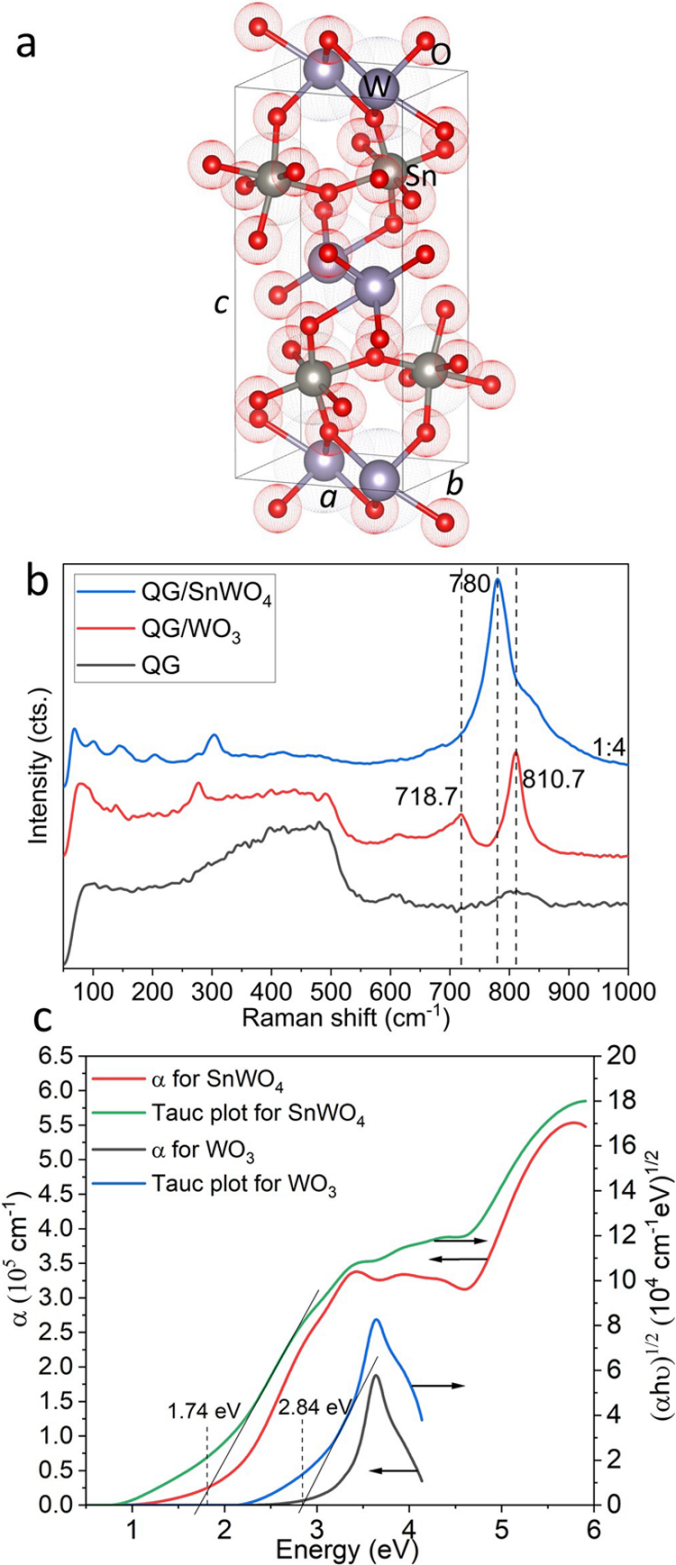
(a) Orthorhombic crystal structure of α-SnWO_4_ crystallized at 450 °C. Gray, blue, and red atoms represent W, Sn, and O. (b) Raman spectra of 110-nm-thick WO_3_ and 90-nm-thick α-SnWO_4_ films on n-Si substrates, their (c) absorption coefficient (α) and Tauc plots as a function of photon energy. The samples were prepared by oxidation of 50-nm-thick W films at 500 °C and further stannation at 450 °C.

A pristine tungsten film exhibits a cubic crystal structure with the (110) reflection at 40.13°, as shown by X-ray diffraction (XRD) patterns (Figure S3a). The cubic structure of W films transforms into a monoclinic WO_3_ phase (PDF 01-072-0677) after annealing at 500 °C in air. Detailed structural comparison of a WO_3_ film with the standard diffraction pattern is shown in Figure S3a. This crystal structure was also identified in magnetron-sputtered WO_3_ films.^31,32^ Heating in vacuum of WO_3_ films in the presence of SnCl_2_ salt leads to their transformation into α-SnWO_4_ films.^10^ The initial thickness of a W film increases accordingly after oxidation and stannation depending on the temperature; phase conversion is accompanied by a change in the unit cell volume (Figure S3b). For instance, the thickness of the 200 nm W film is increased to 290 nm WO_3_ film (30% at 500 °C), 450 nm SnWO_4_ film (55% at 500 °C), and 340 nm SnWO_4_ film (41% at 550 °C). A higher temperature of 550 °C tends to create conditions for fast in- and out-diffusion of SnCl_2_ and WCl_6_ vapors that do not allow for settling in the film compared to 500 °C. Therefore, the thickness of the film crystallized at a higher temperature is less than that for the film prepared at a lower temperature. Naturally, α-SnWO_4_ exhibits an orthorhombic phase according to the standard diffraction pattern (PDF 01-070-1049). Figure 1a shows the elementary crystal structure for an orthorhombic phase of α-SnWO_4_. Detailed structural comparison of the α-SnWO_4_ film with the standard diffraction pattern is shown in Figure S4.

To complement XRD characterization, Raman spectra (Figure 1b) were obtained, showing WO_3_ signals with maximum peak positions at 718.7 and 810.7 cm^–1^ and α-SnWO_4_ signals at 780 cm^–1^. The XRD pattern and Raman spectra for α-SnWO_4_ and WO_3_ are similar to previous reports on detailed structural studies.^33^ Optical properties of the SnWO_4_ films were studied by spectroscopic ellipsometry. The absorption coefficient (α) and Tauc plot of α-SnWO_4_ are compared with those of WO_3_ (Figure 1c). Similar to other well-established materials (CIGS, CdTe, InP, perovskite, etc.),^34^ α-SnWO_4_ exhibits α over the whole shown spectral range ∼10^5^ cm^–1^ at 2.4 eV that is promising with respect to the potential charge-carrier generation due to a suitable band gap (*E*_g_). The indirect band gaps determined by the Tauc plots for α-SnWO_4_ and WO_3_ are 1.74 and 2.8 eV, which are consistent with literature reports.^8,11,35^

**Figure 2:**
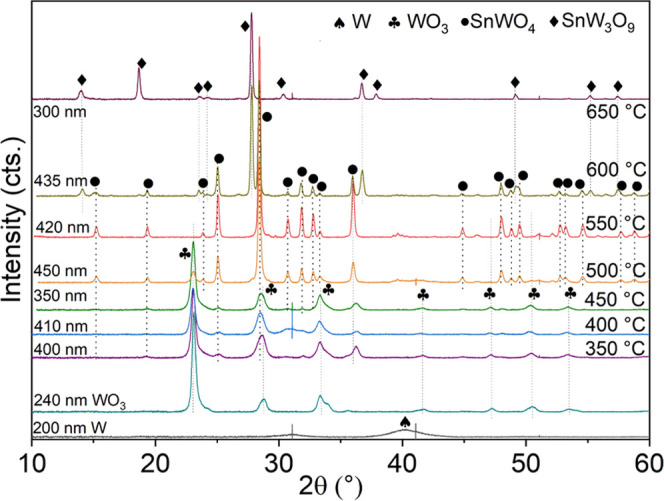
XRD patterns of the films prepared on n-Si substrates in the temperature range from 350 to 650 °C. See also the Raman spectra of the films in Figure S9.

XRD patterns of the samples clearly show that the full crystallization of α-SnWO_4_ depends on the thickness of the WO_3_ films and annealing temperatures (Figure S5). The films thicker than 280 nm feature the WO_3_ secondary phase, as indicated by a (002) reflection at 23.11° (Figure S5a), while the films thinner than 160 nm are composed of only the α-SnWO_4_ phase. This means that during crystallization at 450 °C, chosen as an optimal temperature by Zhu et al.,^10^ Sn cannot diffuse through the whole film hindered by the thickness of WO_3_ films. The discrepancy with that report may be attributed to differences in the experimental configuration affecting the crystallization conditions, for instance, the temperature calibration, tube diameter, powder positioning, boat geometry, etc. Therefore, variation of temperature was done to study the crystallization of the α-SnWO_4_ films in the furnace (Figure 2). In this report, the optimum preparation conditions were found to be 550–600 °C, as shown below.

A series of WO_3_ thin films were heated with SnCl_2_ powder at different temperatures (Figure 2). The WO_3_ films gradually transform into SnWO_4_ between 350 and 550 °C, depending on the thickness. An intensive reflection of a WO_3_ phase at 23.05° (002) is still present for the thicker samples when the temperature is increased up to 500 °C (Figure S5b) and a slight reflection at 550 °C (Figure S5c). At these temperatures, two phases coexist on thick films: WO_3_ and SnWO_4_. An indication of the SnW_3_O_9_ phase can be already detected starting from 500 °C as a small reflection at 27.79° (002) for the thickest samples. This reflection significantly increases with a further temperature increase to 650 °C (Figure 2). At this temperature, complete transformation into the SnW_3_O_9_ phase takes place (Figure S6). The Raman spectra confirm the XRD results (Figure S7), showing how different phase combinations are obtained with a variation of temperature (Figure 2). The thickest single-phase 420 nm thick SnWO_4_ film is achieved at 550 °C. Pure SnWO_4_ films of 160, 330, and 350 nm are obtained at 450, 500, and 600 °C. A reaction furnace setup allowing further increase of the SnCl_2_ concentration could lead to thicker SnWO_4_ films, up to the micrometer range; however, this is out of the scope of the current work. EDX mapping of the film cross sections reveals a homogeneous elemental distribution for films prepared at 500 and 550 °C (Figure S8). Samples prepared at 600 °C are composites of SnWO_4_ and SnW_3_O_9_.

**Figure 3:**
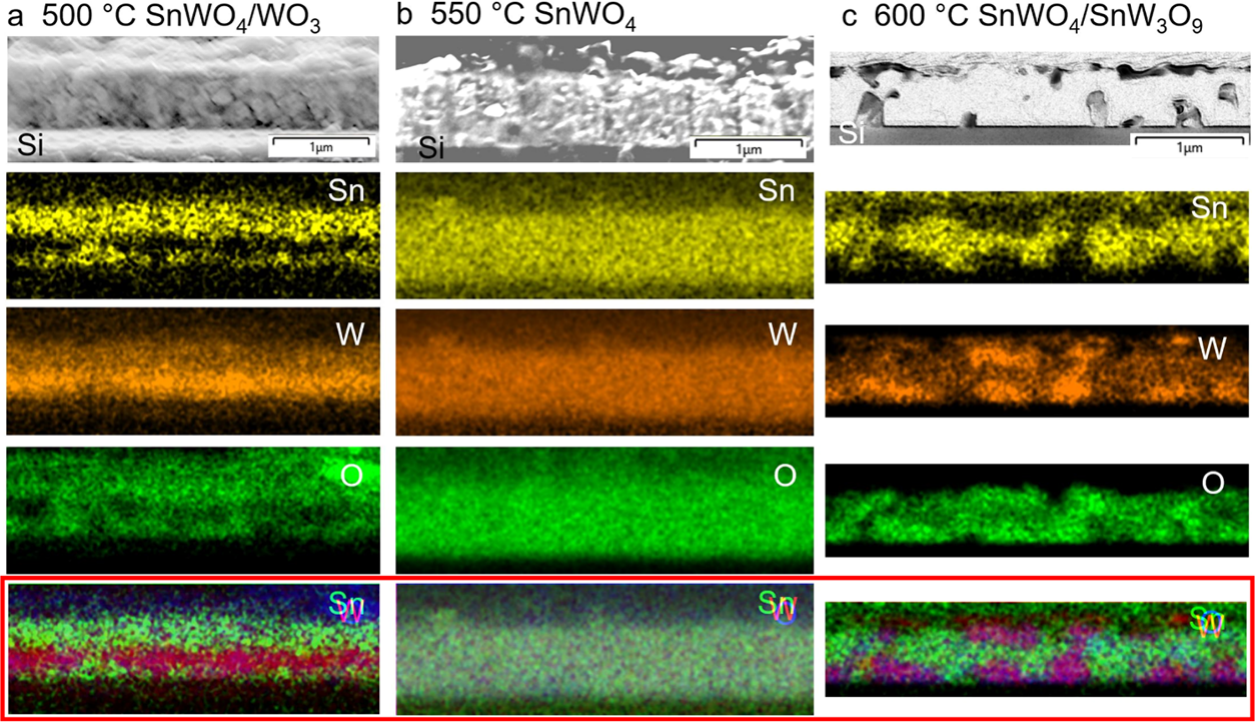
SEM and EDX images for (a) SnWO_4_/WO_3_, (b) SnWO_4_, and (c) SnWO_4_/SnW_3_O_9_ films on n-Si prepared at 500, 550, and 600 °C. Colors of the elements: Sn—yellow, W—orange, and O—green. Below in the red frame are the overlaid mappings: Sn—green, W—pink, and O—violet.

Further insight into the diffusion of Sn into WO_3_ and the formation of SnWO_4_ and SnW_3_O_9_ phases at different temperatures can be obtained from SEM/EDX cross-sectional mappings. The film synthesized at 500 °C consists of a SnWO_4_/WO_3_/SnWO_4_ multilayer, i.e., pure crystallization of SnWO_4_ is not possible (Figure 3a). Sn diffuses easily into WO_3_ from the top as well as from the bottom, since the ionic radius of Sn is 0.145 nm significantly smaller than the lattice parameters of WO_3_: *a* = 0.731 nm, *b* = 0.7603 nm, and *c* = 0.7713 nm.^36^ The reaction of formation of SnWO_4_ can be written as 4WO_3_ + 3SnCl_2_ → 3SnWO_4_ + WCl_6_↑. Voids and defects at the bottom of the film enhance the penetration of the SnCl_2_ vapor into the film as well as the diffusion of the byproduct WCl_6_ out of the film. Thus, the reaction starts at the top and at the bottom of the film, which results in bands of higher Sn concentration toward the film surface and toward the Si substrate (yellow bands in Figure 3a) on the EDX maps. In addition, the O-poor region clearly indicates WO_3_ (three O atoms to one W atom) compared to O reach region of SnWO_4_ (four O atoms to one W atom). An intermediate WO_3_ band can be clearly seen between the SnWO_4_ bands. The full conversion into SnWO_4_ is realized at 550 °C, where Sn, W, and O are homogeneously distributed across the film (Figure 3b). Larger grains of SnWO_4_ are formed, compared to those of the precursor WO_3_, as evidenced by the narrower full width at half-maximum (FWHM) of the most intensive peak for (121)-SnWO_4_ with respect to (020)-WO_3_ (Figure 2). This would point to an increased mobility of Sn as a main factor leading to a full reaction with WO_3_ at a higher temperature. A possible mechanism for the Sn and W diffusion could then be similar to the interdiffusion of Cu and In in a CuInS_2_ (CIS) film,^37^ where Cu enrichment of Cu-poor CIS films leads to grain growth. This was similarly observed by decreasing the FWHM of the (112)-CIS peak during the recrystallization. Furthermore, as soon as Sn is incorporated into WO_3_, there would be unequal diffusion of Sn and W through the vacancies according to the Kirkendall effect.^38^ This could be one reason for different crystallization mechanisms at 500, 550, and 600 °C in Figure 3. The formation of the SnWO_4_ phase is expected at elevated temperatures. However, at 600 °C, a secondary SnW_3_O_9_ phase is formed in addition to SnWO_4_, on top of the film as well as toward the Si substrate (Figures 3c and S10). Sn leaves the film bulk due to its higher mobility, resulting in the crystallization of a Sn-poor phase: SnW_3_O_9_. This is clearly seen as highly concentrated W and O crystals oriented variously in space, which indicate higher volatility of SnCl_2_ compared to that of heavier WCl_6_. A balance between in-diffusion of SnCl_2_ and out-diffusion of WCl_6_ in the films is achieved at 550 °C, favorable to the formation of α-SnWO_4_. EDX cross-sectional mappings of the films in high resolution for quantitative visualization are shown in Figure S10. These results confirm the phase compositions of SnWO_4_/WO_3_, SnWO_4_, and SnW_3_O_9_/SnWO_4_ phases at 500, 550, and 600 °C, as revealed by the XRD data shown in Figure 2.

**Figure 4:**
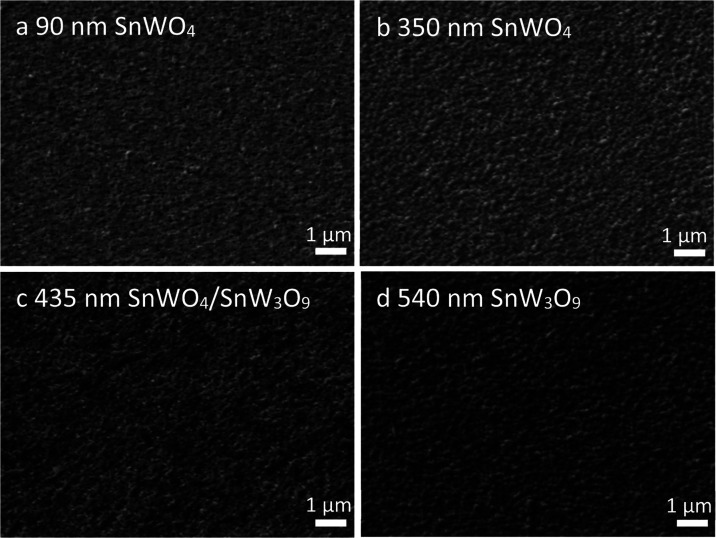
SEM images for (a) 90 nm SnWO_4_, (b) 350 nm SnWO_4_, (c) 435 nm SnWO_4_/SnW_3_O_9_, and (d) 540 nm SnW_3_O_9_ films on n-Si prepared at 600 °C. SEM cross-sectional images for SnWO_4_ and SnW_3_O_9_ are shown in Figure S12.

Tin tungstate films prepared with various thicknesses at 600 °C exhibited SnWO_4_ phase up to a thickness of about 350 nm (Figure S5d). The film with the thickness of 435 nm shows the presence of an additional hexagonal phase of SnW_3_O_9_ (PDF 01-086-0628).^39,40^ Further increase in thickness led to the preferential formation of SnW_3_O_9_, as evidenced in the diffractograms for 540, 590, and 640 nm thick films (Figures S5d and S6). These results are confirmed by Raman spectra, where the Raman peak at 780 cm^–1^ disappears at a higher film thickness (Figure S7). This means that the rate and amount of Sn diffusion significantly influence on the crystallization of the films. Full crystallization of SnWO_4_ for the thick films might be possible with higher amounts of SnCl_2_ powder present. However, further addition of powder and subsequent heating experiments at 600 °C did not result in the transformation of SnW_3_O_9_ into SnWO_4_ (Figure S11). Therefore, the formation of SnW_3_O_9_ should be avoided if the objective is to get pure SnWO_4_. SEM images of these films confirm the increase in the crystallite size when the thickness increases from 90 to 350 nm (Figure 4a,b). The transition into SnW_3_O_9_ is detected for a 435-nm-thick film, where large platelets with fine particles are seen on the surface (Figure 4c). Bigger particles with crystalline SnW_3_O_9_ properties are confirmed by growing (200) reflection intensity and its decreasing FWHM (Figures S5d and 4c,d).

### TSPV Performance

**Figure 5:**
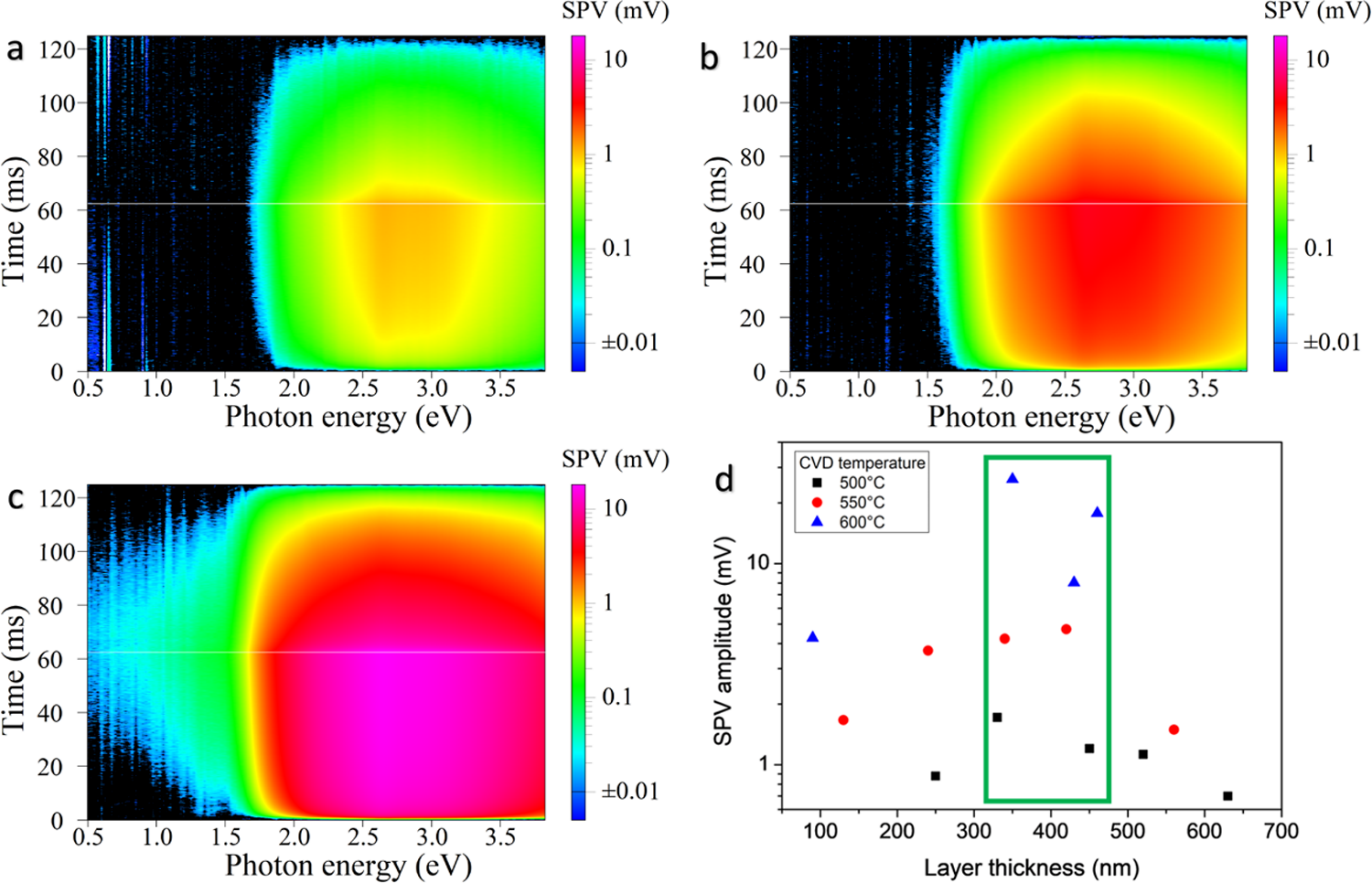
TSPV spectra of ∼460 nm thick samples prepared at CVD temperatures of (a) 500 °C, (b) 550 °C, and (c) 600 °C and maximum SPV amplitude of TSPV spectra of samples of different layer thicknesses and (d) prepared at different CVD process temperatures. The optimum film-thickness region is shown with the green frame to be in the range of 300–450 nm.

TSPV measurements were performed on a series of tin tungstate phase samples with layer thicknesses between 90 and 630 nm prepared at chemical vapor deposition (CVD) process temperatures of 500, 550, and 600 °C, and example spectra for each temperature are shown (Figure 5a–c). All spectra show a positive SPV signal above the band gap, indicating the n-type behavior of the material. The maximum SPV amplitude (Figure 5d) was found for all process temperatures between 300 and 450 nm, indicating an optimum layer thickness for photoelectrodes of that material in the same range.

**Figure 6:**
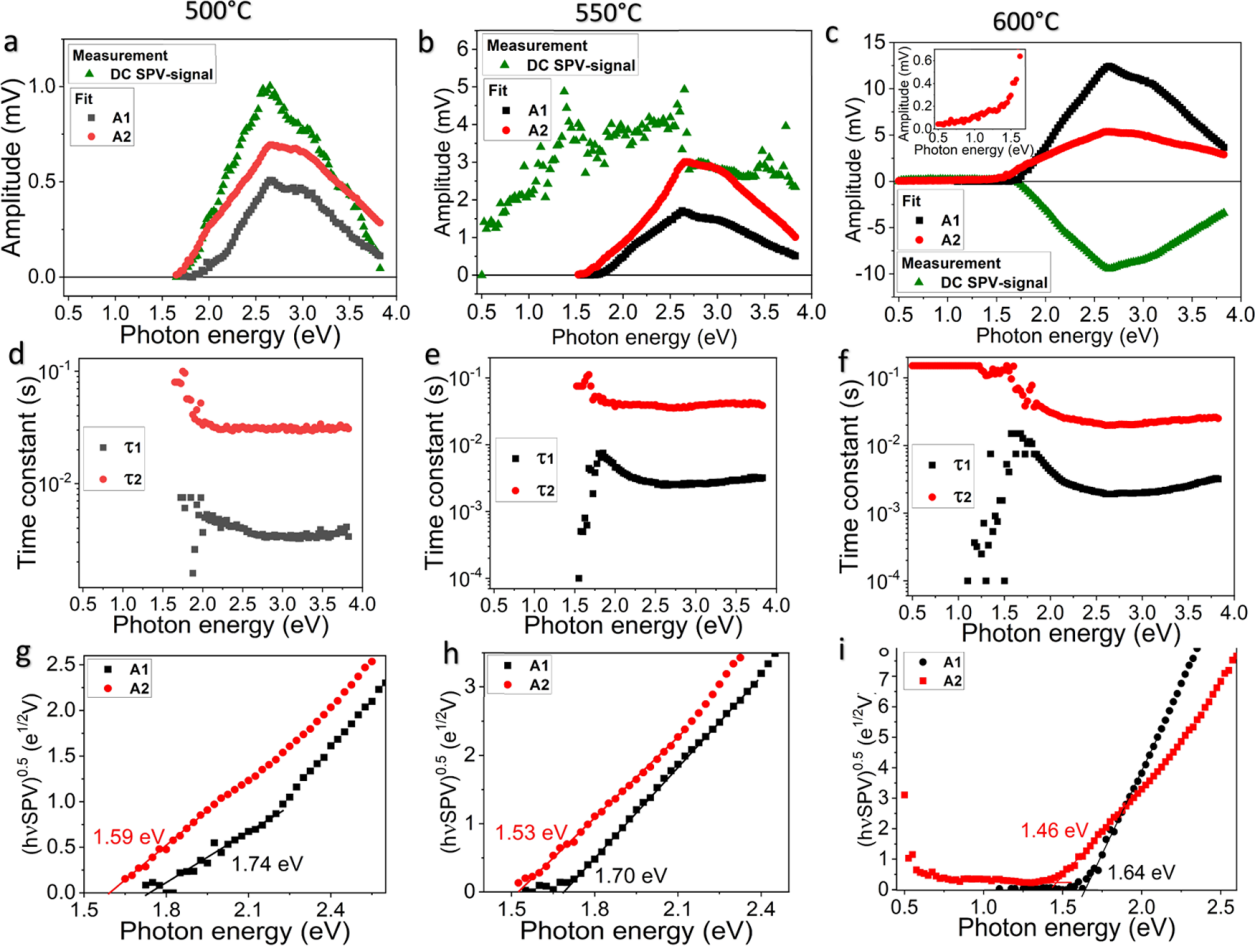
(a–c) Amplitudes and (d–f) characteristic times obtained by fitting of TSPV spectra of samples prepared at CVD temperatures of 500, 550, and 600 °C as well as derived Tauc plots (g–i). Also shown are the DC components of the SPV signal (a–c).

The individual transients that constitute the spectra were fitted in the light-on phase (*t* = 0–62.5 ms), each with a sum of two exponential functions. Each exponential function represents charge-carrier transport processes with a characteristic time (τ_*i*_). The fitting yielded for the two exponential functions amplitudes (*A*_*i*_) and characteristic times, as shown in Figure 6a–c,d–f, respectively. The trends in the characteristic times and amplitudes look similar for all samples except for the exponential function 2 of the 600 °C sample. For this sample, an additional process at energies below 1.5 eV was found (that process was also described by the second exponential function). The amplitude of this additional process is shown in the inset of Figure 6c. The low onset energy of that process around 0.5 eV is in good agreement with the band gap of SnW_3_O_9_. In the graphs showing the amplitudes, in addition, also the direct current (DC) component of the SPV signal was plotted. The DC component describes the processes with a charge-carrier dynamics that is too slow to follow the excitation frequency. For the 500 and 600 °C samples, the DC component followed a trend that is correlated with the amplitude spectra, while for the 550 °C sample, the DC component was for energies below 2.7 eV unrelated to the excitation during the spectral measurement. In this range, the signal was determined by discharging of the sample, while for energies above 2.7 eV, the signal deviated abruptly from the prior discharging trend. This is constated exactly at the band gap of WO_3_, indicating that the charge-carrier transport dominating the DC component is taking place in WO_3_. The fact that already at the band gap the charging changes immediately points toward a high photon flux indicates that the WO_3_ layer in question is located at the surface where the exciting illumination was not already reduced by absorption in SnWO_4_. The fact that the fluctuation in the DC component also vanished after the onset of the absorption in WO_3_ indicates that the whole signal of the DC component originates at the surface and therefore that the discharging that takes place below 2.7 eV is a discharging of surface states at or nearby the WO_3_ layer. For the 500 °C/600 °C sample, the positive/negative DC component that was correlated with the amplitude of process 1 indicates an accumulation of holes/electrons close to the surface.

In the small signal case, the SPV signal can be assumed to be in good approximation linearly dependent on the photon flux of the excitation. To get a better idea about the origin of the process that led to contributions of the SPV signal described by the exponential functions, the amplitudes found by the fit were normalized against the photon flux, and the square root of the product of normalized SPV amplitude and excitation energy was plotted (Figure 6g–i). These plots are helpful to find the indirect transition energy related to the processes identified. For the 500 °C sample, transition energies of 1.74 and 1.59 eV were found for processes 1 and 2. The transition energy enabling the charge-carrier transport process 1 is identical to the band gap found for SnWO_4_ and can therefore be attributed to the band–band transition in SnWO_4_. The transition energy of the first process was for all samples close to the band gap of SnWO_4_ but decreasing in energy with increasing temperature. The transition energy of the second process was for all samples 170–180 meV below the first transition energy and decreased with increasing temperature. Also, considering the n-type behavior of the samples, the most likely origin of that process is the transition between the valence band and donor states.

**Figure 7:**
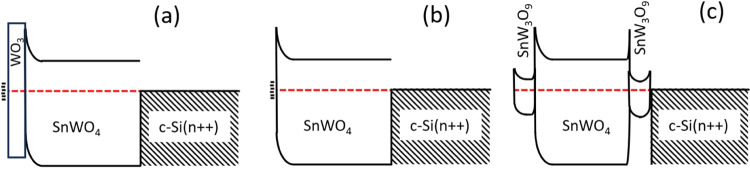
Band schemes derived from TSPV measurements for the samples prepared at (a) 500 °C, (b) 550 °C, and (c) 600 °C.

The band schemes were derived from the analyzed SPV measurements (Figure 7). For the 500 °C sample (Figure 7a), an upward band bending led to separation of holes toward the surface leading to a positive SPV signal. The deep defects at the surface led to long trapping times for holes, resulting in a SPV DC component with a positive sign that followed the faster signal components (processes 1, 2). There was no signature for the nonstoichiometric region in the SnWO_4_ layer close to the substrate. For the 550 °C sample (Figure 7b), the DC component was uncorrelated to the faster processes at lower excitation energy because the layer responsible for the signal was practically not accessible for charge carriers generated in the SnWO_4_ layer. The DC component changed its character only when charge carriers were photogenerated in the WO_3_-surface layer. For the 600 °C sample, the slow processes that led to the negative DC component were mainly due to electrons trapped near the surface SnW_3_O_9_ phases, after being photogenerated in a neighboring region without the SnW_3_O_9_ surface layer and being separated over the SnWO_4_/SnW_3_O_9_ heterojunction at the interface between those regions (see for comparison EDX cross sections of the 600 °C sample in Figure 3c). Therefore, the band scheme for the 600 °C sample is in parts of the sample given by Figure 7a, while it is in the neighboring region given by a version of Figure 7c with one or both SnW_3_O_9_ layers shown.

### XPS Studies

**Figure 8:**
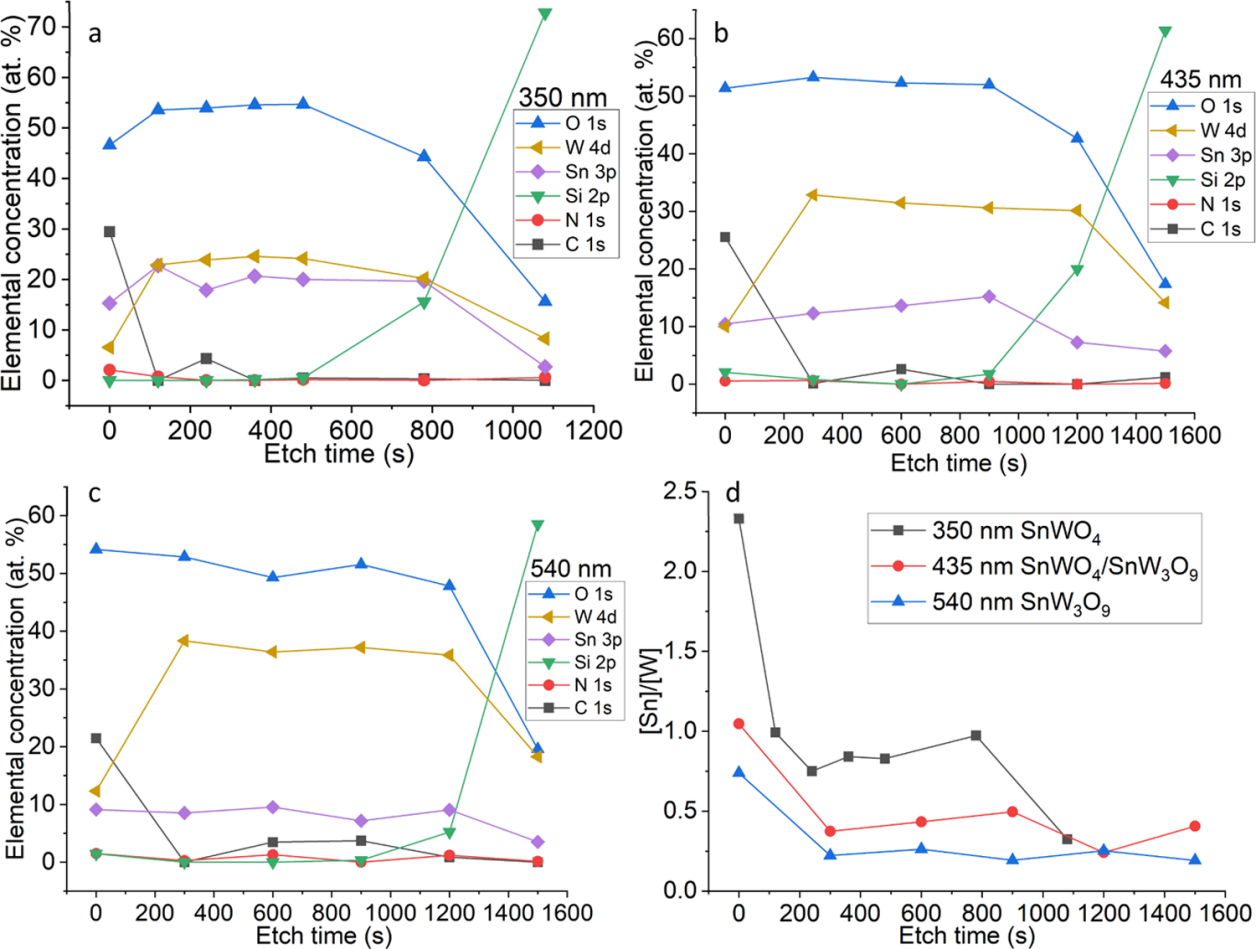
XPS spectral evolution of (a) 350 nm thick SnWO_4_, (b) 435 nm thick SnWO_4_/SnW_3_O_9_, and (c) 540 nm thick SnW_3_O_9_ films crystallized at 600 °C as a function of etch time. (d) [Sn]/[W] ratio as a function of the etch time.

The X-ray photoelectron spectroscopy (XPS) spectra for samples of different thicknesses prepared at 600 °C were obtained at increasing etch depths to investigate the elemental distribution in the films (Figure 8). All films contain carbon on the surface, as is clear from the higher concentrations in the beginning of the etching process. This carbon contamination comes from the environment, e.g., plastic substrate holder, the rest in the muffle oven that can be deposited during crystallization, etc. After removal of the surface carbon, a clear one-to-one relation between Sn and W is evidenced, which confirms crystallization of the 350 nm thick SnWO_4_ film (Figure 8a). Increasing thickness up to 435 nm results in about three times higher W concentration compared to Sn concentration (∼10 to ∼30 atom %), indicating the formation of the mixture SnWO_4_/SnW_3_O_9_ (Figure 8b). At higher thickness up to 540 nm, about four times higher W concentration with respect to Sn (∼10 to ∼40 atom %) is evidenced, due to the formation of SnW_3_O_9_ (Figure 8c). The concentration of O remains almost constant over the thickness for all films (∼45 to ∼55 atom %). The last etching phase shows a maximum Si composition of about 60–70 atom %, as the substrate is increasingly probed, due to the removal of Sn, W, and O. The [Sn]/[W] ratio quantitatively shows (Figure S5d) the evolution of phase composition from SnWO_4_, SnWO_4_/SnW_3_O_9_, and SnW_3_O_9_ phases during the etch process, from the surface and into the bulk of the film (Figure 8d). The [Sn]/[W] ratio for the 350 thick film is close to 1 corresponding to pure SnWO_4_. For the 435 thick film, the ratio is about 0.4, due to the presence of a mixture of SnWO_4_/SnW_3_O_9_. For the 540 thick film, the ratio is close to 0.25, corresponding to SnW_3_O_9_.

### PEC Performance

**Figure 9:**
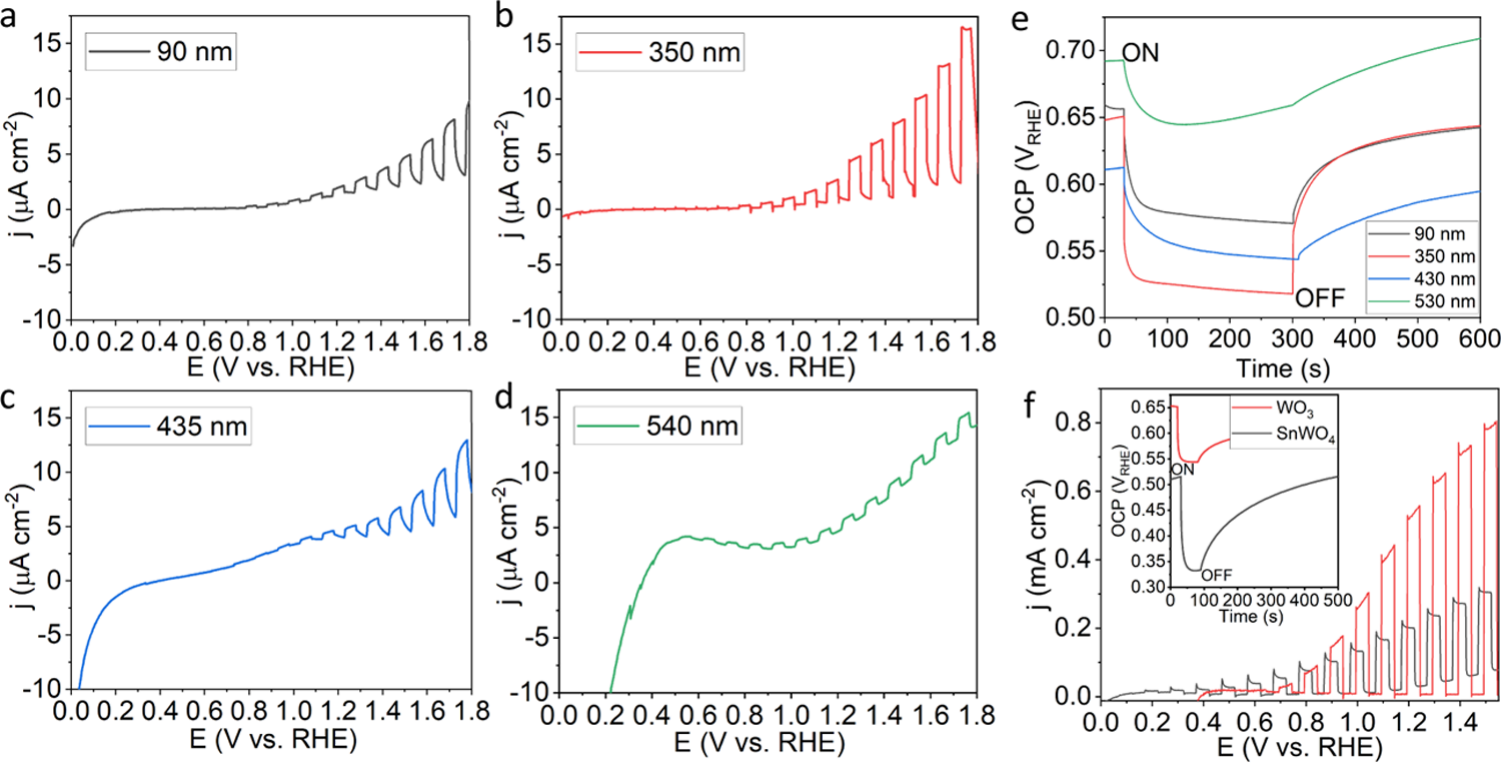
Linear scan voltammetry for (a) 90 nm SnWO_4_, (b) 350 nm SnWO_4_, (c) 435 nm SnWO_4_/SnW_3_O_9_, and (d) 540 nm SnW_3_O_9_ thick films on n-Si in 0.5 M Na_2_SO_4_ and (e) their OCP. (f) Comparison of the linear scan voltammetry for a 400 nm WO_3_ film prepared at 500 °C in air and transformed into a 520 nm SnWO_4_ film at 450 °C with SnCl_2_ powder in vacuum on FTO substrates in 0.5 M Na_2_SO_4_.

An increase in the SnWO_4_ film thickness from 90 to 350 nm results in a clear improvement of the photocurrent density, *j* (Figure 9a–d). The 350 nm film showed the best photocurrent densities. The mixture SnWO_4_/SnW_3_O_9_ showed a decrease in the photocurrent response due to the contribution of SnW_3_O_9_. The main reason for *j* decrease is a junction formation between SnWO_4_/SnW_3_O_9_ and low minority charge carrier diffusion length *L*_D_, resulting in limited lifetime within the thicker film.^7^ All films on Si exhibit *j* in the range of 1–15 μA cm^–2^, significantly lower than the films prepared on FTO. The open-circuit potential (OCP) measurements agree with the photocurrent results. The 90 nm SnWO_4_, 350 nm SnWO_4_, 435 nm SnWO_4_/SnW_3_O_9_, and 540 nm SnW_3_O_9_ thick films on n-Si resulted in photovoltage (*V*_ph_ = *V*_OC,dark_ – *V*_OC,light_) values of 0.10, 0.14, 0.07, and 0.05 V (Figure 9e). The highest dark current is observed for the SnW_3_O_9_ film, and the lowest for SnWO_4_ that indicates the lowest resistivity for SnW_3_O_9_ and highest resistivity for SnWO_4_ (Figure S13). The mixture of SnWO_4_/SnW_3_O_9_ exhibited an intermediate dark current. This observation proves that SnWO_4_ and SnW_3_O_9_ are not formed as separate layered films, intermixing instead through the film’s bulk. In the case of a layered film formation, the resistivity of SnWO_4_ would remain at least as high as that observed for the thinnest 90 nm SnWO_4_ film.

The PEC performances for WO_3_ and SnWO_4_ films are compared in Figure 9f. The WO_3_ film shows a higher *j* at positive potentials. The photocurrent densities *j*, measured at 1.21 V_RHE_, were 0.17 and 0.53 mA cm^–2^ for SnWO_4_ and WO_3_, respectively. However, the SnWO_4_ film shows a *j* response starting at 0.07 V_RHE_ and at 0.60 V_RHE_ for WO_3_. Lower onset potential is an indication for lower energy needed to drive the PEC reaction. As discussed, SnWO_4_ has more suitable conduction and valence band positions that straddle both HER and OER potentials.^8^ With increasing potential, the dark current for SnWO_4_ increases, compared to WO_3_ (Figure 9f). This has been attributed to the formation of SnO_2_ and WO_3_.^35^ Further cycling leads to a decrease in *j* and dark current due to self-passivation, as SnO_2_ serves as a hole-blocking layer (Figure S14). Stabilization efforts in different pH values and with the NiO_*x*_ protection-coating have been reported in the literature.^8,21^ It was found that SnWO_4_ does not degrade in acidic up to neutral conditions at pH values of 2, 5, and 7 but in alkaline solution starts to decompose at pH 9 and 13 upon illumination. Furthermore, 24 h of testing led to the dissolution of NiO_*x*_ in the electrolyte. In this work, in an attempt to stabilize SnWO_4_, an overlayer of 20 nm of TiO_2_ was deposited by atomic layer deposition (ALD). PEC testing showed a significant *j* decrease and still the oxidation of SnWO_4_ (Figures S15 and S16). The chronoamperometry curve shows a positive dark current in the beginning, which decreases over time. Other possible protection-coatings should be explored to protect the surface of SnWO_4_ without reducing its activity.

Interestingly, the measured *V*_ph_ = 0.185 V for SnWO_4_ is higher than *V*_ph_ = 0.110 V measured for WO_3_, in an inverse relationship to the measured photocurrents. For the wider *E*_g_ (SnWO_4_: 1.74 eV and WO_3_: 2.8 eV, Figure 1d), higher *V*_ph_ is expected. This may be explained by better charge-carrier separation in the film bulk for SnWO_4_, accompanied, however, by weaker charge transfer from the film across the semiconductor/electrolyte junction. A smoother surface (smaller surface area) for WO_3_ on FTO can also be a factor that contributes to reducing the photon flux at the surface and producing a lower photovoltage. This observation is clear from the transmittance spectra for WO_3_ that shows interference fringes due to more surface homogeneity (Figure S17).

The PEC performance of SnWO_4_ was studied on top of different substrates. It has been found that *j* is significantly lower on Si than on TiN:O and FTO (Figures 9 and S18). The morphology of the films is rougher and therefore *j* is higher due to the higher surface area in these substrates (Figures S19 and S20). LSV curves for SnWO_4_ films show that the films are not stable after cycling compared to the more stable WO_3_ on FTO substrates (Figures S21 and S22). Furthermore, crystallization depends on the type of the substrate. A dendritic structure was observed on a quartz glass substrate for the 100 nm thick SnWO_4_ film at 450 °C (Figure S23). Such kind of oak tree leaf structures crystallize as a result of the attachment of Sn, W, and O atoms to nucleation centers (particles) or inhomogeneities. The film prepared at 600 °C exhibits weak adhesion to the substrate and therefore agglomerates on the surface as spate particles (Figure S24). A homogeneous SnWO_4_ surface tends to grow only on Si compared with quartz, FTO, and TiN/O.

The measured *j* = 0.17 mA cm^–2^ is 100 times lower than the theoretical maximum *j* ∼ 17 mA cm^–2^ under AM1.5G solar irradiation, expected for *E*_g_ = 1.9 eV and considering that 100% of photons are absorbed by SnWO_4_. There have been no reports demonstrating photocurrents beyond 2.0 mA cm^–2^, the maximum experimentally measured *j* being 1.05 mA cm^–2^ at 0 V_RHE_ for SnWO_4_.^41^ This clearly indicates that the quality of the films should be significantly improved. Temperatures higher than 450 °C led to the inhomogeneous crystallization (phase separation and agglomeration) of the films on FTO substrates due to the higher mobility of Sn and different expansion coefficients of WO_3_ and FTO. Therefore, possible strategies for the improvement of coating adhesion should be further investigated.

A comprehensive review on SnWO_4_ for PEC water oxidation has been reported recently.^42^ Different scavengers were used to boost the charge-carrier transport from the semiconductor into the electrolyte to investigate the maximum performance. The summary from all current reports is that the main efforts should be directed to elaboration of protection-coatings that could stabilize the surface of SnWO_4_ against SnO_2_ formation and in turn boost *j*. Furthermore, the generated photovoltage *V*_ph_ = 0.185 V is very low to drive water splitting (1.23 V), even though in theory *E*_g_ = 1.74 eV of SnWO_4_ should be close to optimum. Herein, the main losses are attributed to series resistances, adhesion, defects, low *L*_D_ ∼ 75 nm,^23^ and recombination in the bulk, and at the semiconductor–electrolyte interface. To achieve the maximum theoretical *j* = 17 mA cm^–2^, a film thickness of about 10 μm would be necessary.^23^ This thickness is significantly larger than the minority carrier diffusion length, *L*_D_, which is a main limiting factor considering expected losses at grain boundaries and defects in the structure. This issue remains the main challenge for many ternary metal oxides. Each factor should be further investigated for these materials.^12,42^ Generally, stabilization of any photoelectrode surface for PEC water splitting is an open question that hinders their wide-scale implementation in practice. The main requirement is that the protection-coating should be electrochemically stable, while not hindering *j*, *V*_ph_, and light absorption, and should be cost-effective. Even though SnWO_4_ exhibits a high absorption coefficient α ∼ 10^5^ cm^–1^ (Figure 1c) and a promising *E*_g_ value, allowing absorption of the most visible part of the solar spectra and optimum valence and conduction band positions for the redox reaction of water, the carrier dynamics of the films should be further improved.

## Conclusions

The influence of the temperature and *W* thickness on the crystallization of Sn tungstate films was investigated. A series of WO_3_ thin films were converted into SnWO_4_ by reaction with SnCl_2_ at increasing temperatures. The synthesis of SnWO_4_ and SnW_3_O_9_ can be controlled by the temperature and thickness of the WO_3_ films. High temperatures (500–650 °C) and thicknesses higher than 435 nm led to the formation of the SnW_3_O_9_ phase, while lower thicknesses resulted in single-phase SnWO_4_ films. At lower temperatures (*T* < 450 °C), films of thickness beyond 280 nm still exhibit the WO_3_ phase along with SnWO_4_. However, thinner films crystallize preferentially as SnWO_4_. The presence of the SnW_3_O_9_ phase in the films leads to reduced device performance, i.e., its formation needs to be impeded by controlling the temperature and thickness of the films. The optimal temperatures and thicknesses for the preparation of single-phase SnWO_4_ films with the best transient surface photovoltage (TSPV) and photoelectrochemical cell (PEC) performance are found to be 550–600 °C and 300–450 nm. Further studies should be focused on the preparation of micrometer-thick films with the highest quality to achieve photocurrents closer to the theoretical limit.

## Supporting Information Available

Additional experimental details, photographs of experimental setup, optical images of the films, ellipsometry, XRD, Raman spectra of all compounds, and PEC measurements (PDF)
