# Supplementary material for: Influence of SnWO4, SnW3O9, and WO3 Phases in Tin Tungstate Films on Photoelectrochemical Water Oxidation
Source: ACS Appl Mater Interfaces. 2024 Sep 3;16(36):48565–75. doi: 10.1021/acsami.4c09713 (PMC11403547; doi:10.1021/acsami.4c09713)
Supplement: Supplementary file 1 — am4c09713_si_001.pdf [file am4c09713_si_001.pdf]

Supporting Information

Influence of $SnWO_4$, SnW_3O_9, WO_3 phases in tin tungstate films on photoelectrochemical water oxidation

Farabi Bozheyev,[a-c]* Steffen Fengler,[a] Jiri Kollmann,[a] Daniel Abou-Ras,[d] Nico Scharnagl,[e] Mauricio Schieda[a]*

[a]Institute of Photoelectrochemistry, Helmholtz-Zentrum Hereon GmbH, Max-Planck-Str. 1, 21502 Geesthacht, Germany

[b]Institute of Applied Science and Information Technologies, Baizakov Str. 280, 050042 Almaty, Kazakhstan

[c]National Nanolaboratory, Al-Farabi Kazakh National University, al-Farabi Ave. 71, 050000 Almaty, Kazakhstan

[d]Department Structure and Dynamics of Energy Materials, Helmholtz-Zentrum Berlin für Materialien und Energie GmbH, Hahn-Meitner-Platz 1, 14109 Berlin, Germany

[e]Institute of Surface Science, Helmholtz-Zentrum Hereon GmbH, Max-Planck-Str. 1, 21502 Geesthacht, Germany

*Corresponding authors: farabi.bozheyev@gmail.com and mauricio.schieda@hereon.de

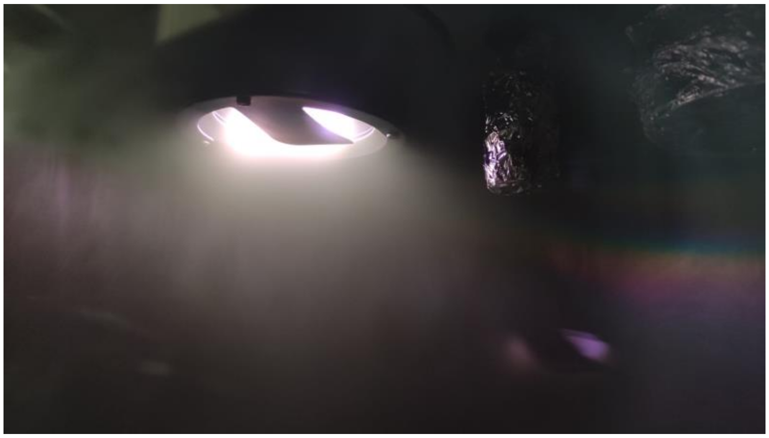

Figure S1. Magnetron sputtering of a W target in an Ar atmosphere. Sputtering rate was 4.5 nm/min at a 100 mA current.

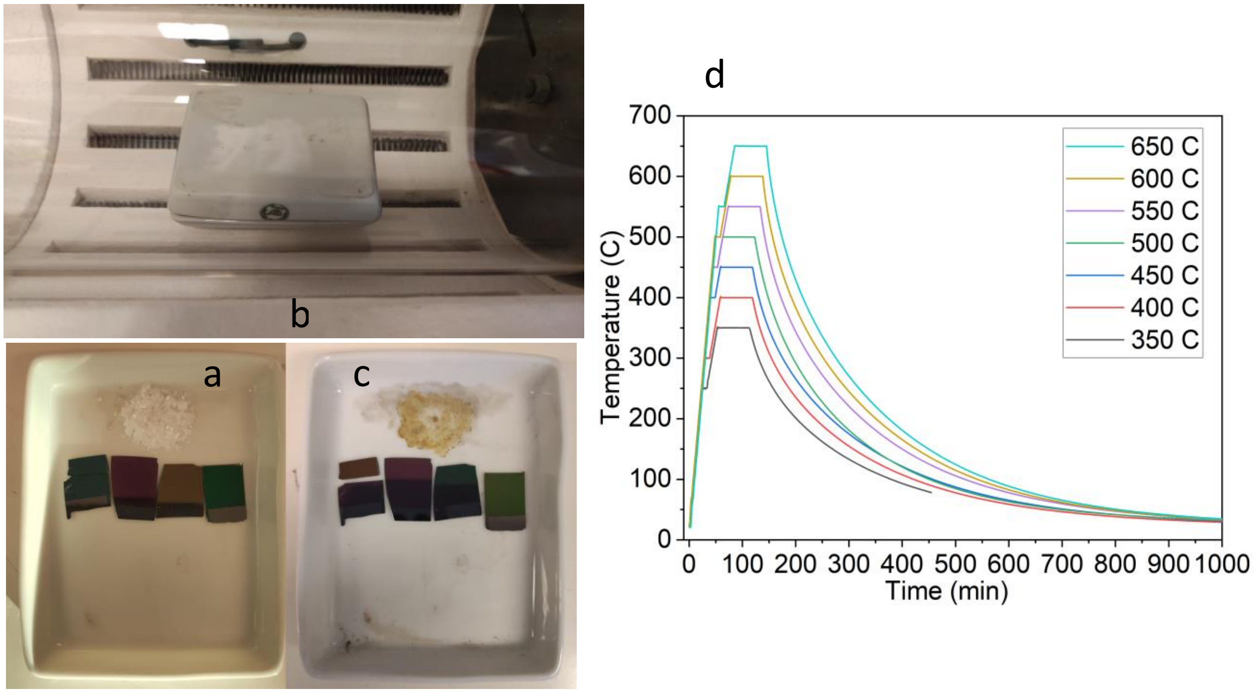

Figure S2. Preparation of $SnWO_4$ in the tube furnace. (a) The WO_3 samples on n-Si with a 5 mg $SnCl_2$ sault. (b) The boat was covered with a similar size of boat from the top and put into a tube furnace. (c) $SnWO_4$ films after crystallization at 450 °C in vacuum. (d) Various crystallization temperatures used to prepare tin tungstate films: before reaching the maximum, the temperature was kept during 10 min to stabilize the temperature ramp. This can be seen as a temperature step.

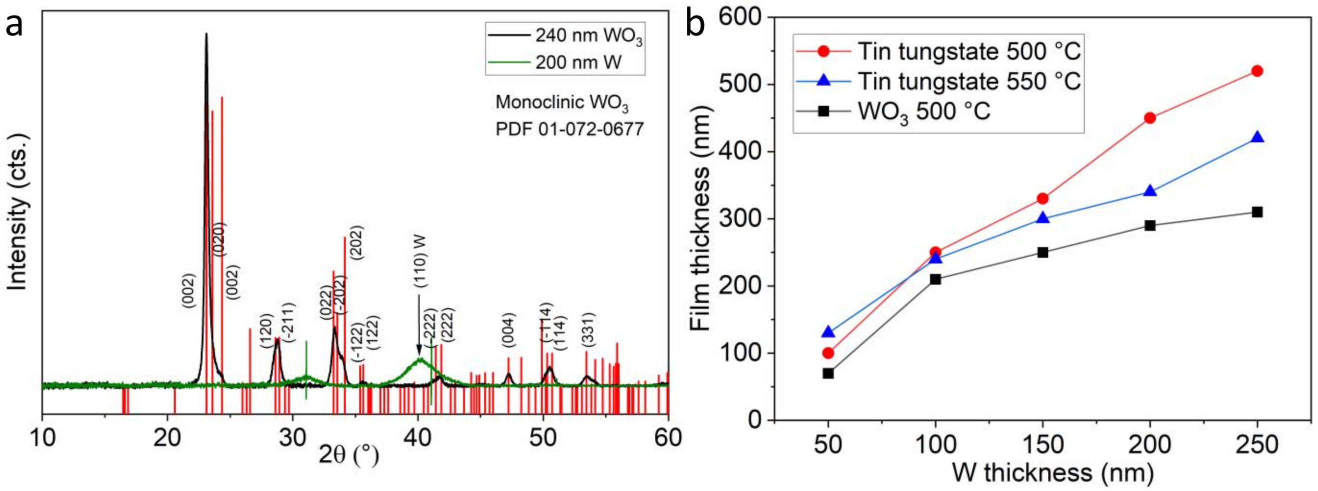

Figure S3. (a) XRD pattern for a 200 nm thick W and 240 nm thick WO_3 film prepared on a Si substrate compared with the PDF 01-072-0677 standard diffraction pattern for WO_3. (b) (b) Thickness of WO_3 and tin tungstate films grown at 500 °C and 550 °C, for a given thicknesses of the starting W film.

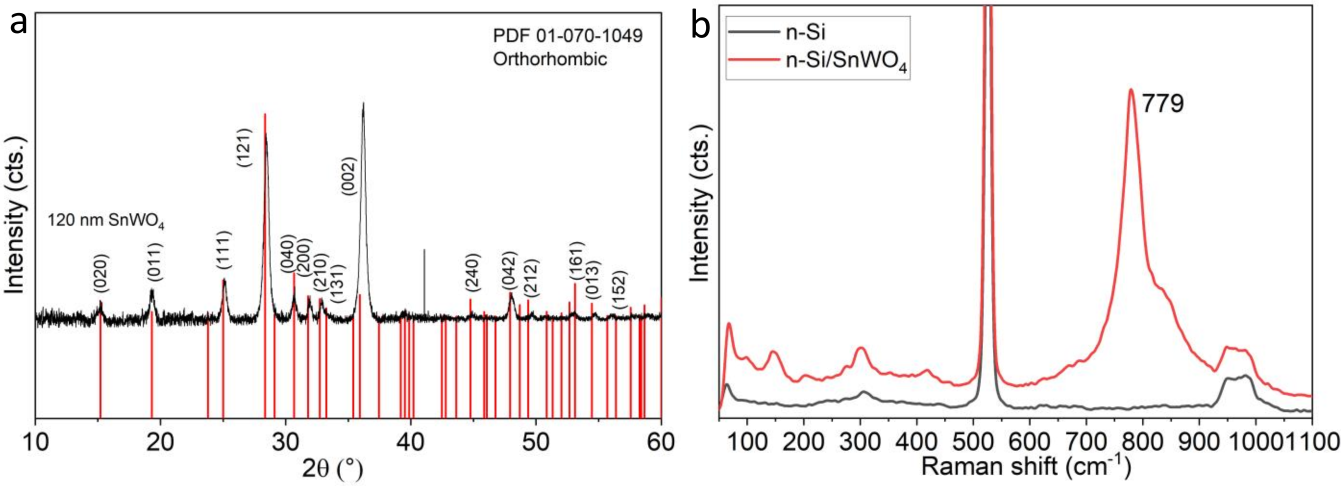

Figure S4. (a) XRD pattern for a 120 nm thick $SnWO_4$ film crystallized at 450 °C on n-Si substrate compared with the PDF 01-070-1049 standard diffraction pattern and (b) its Raman spectra.

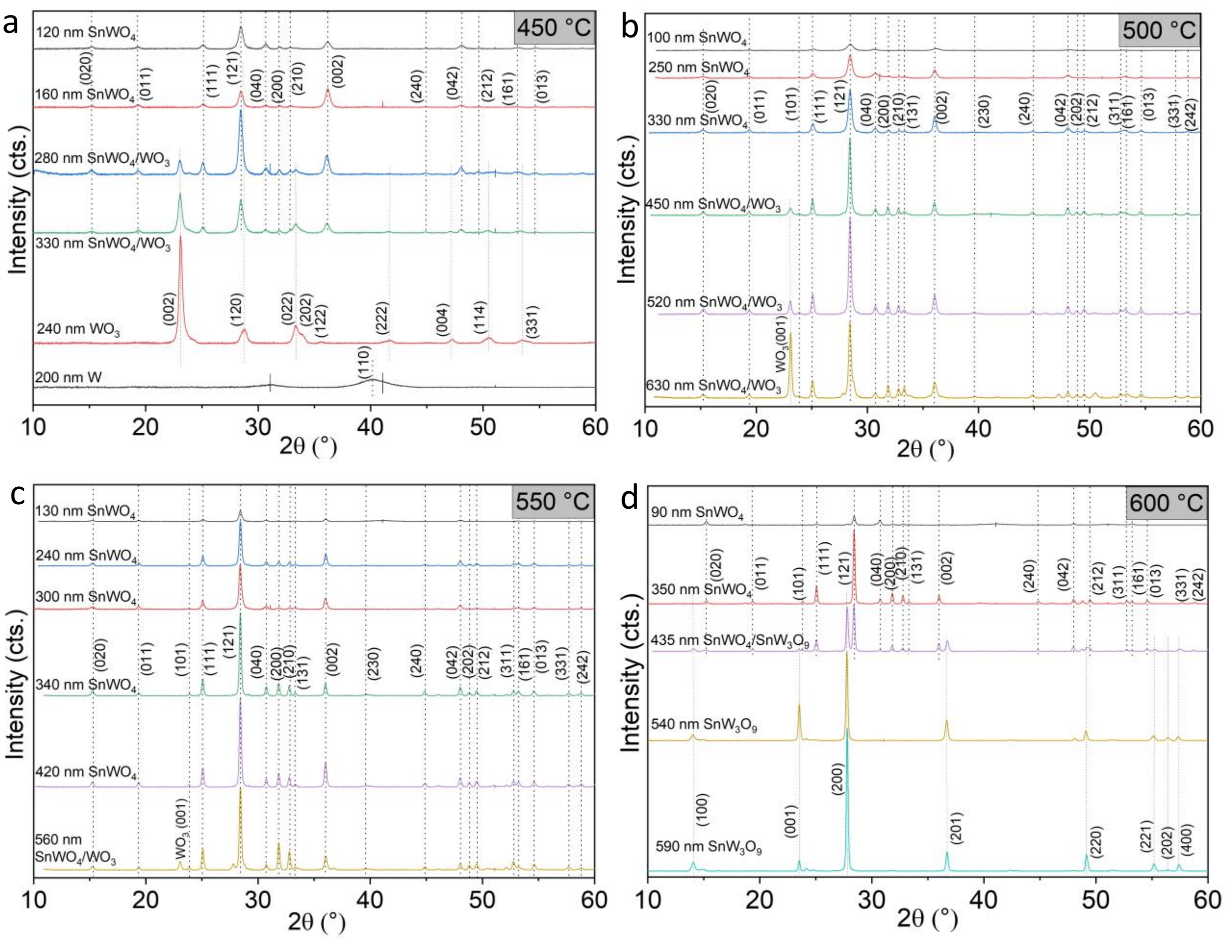

Figure S5. XRD patterns of the different thick tin tungstate films crystallized at 450 °C (a), 500 °C (b), 550 °C (c), and 600 °C (d) on n-Si substrates.

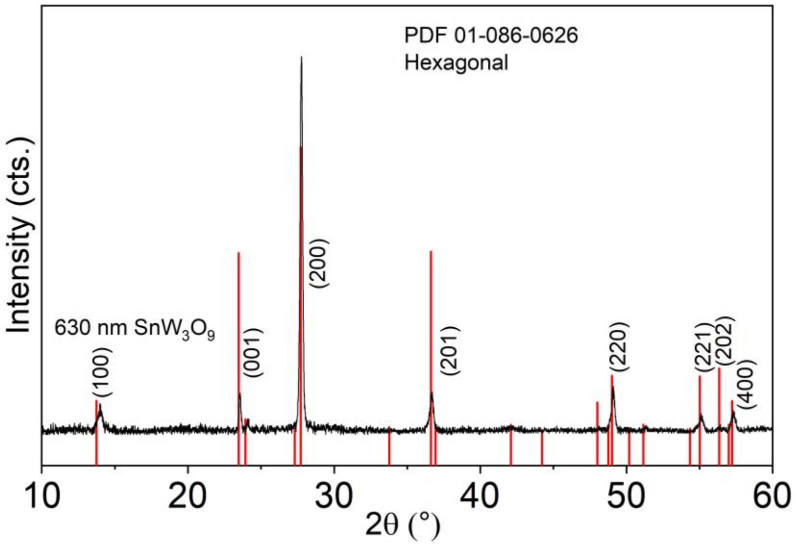

Figure S6. XRD pattern for a 630 nm thick SnW_3O_9 film prepared on n-Si substrate at 600 °C compared with the PDF 01-086-0626 standard diffraction pattern.

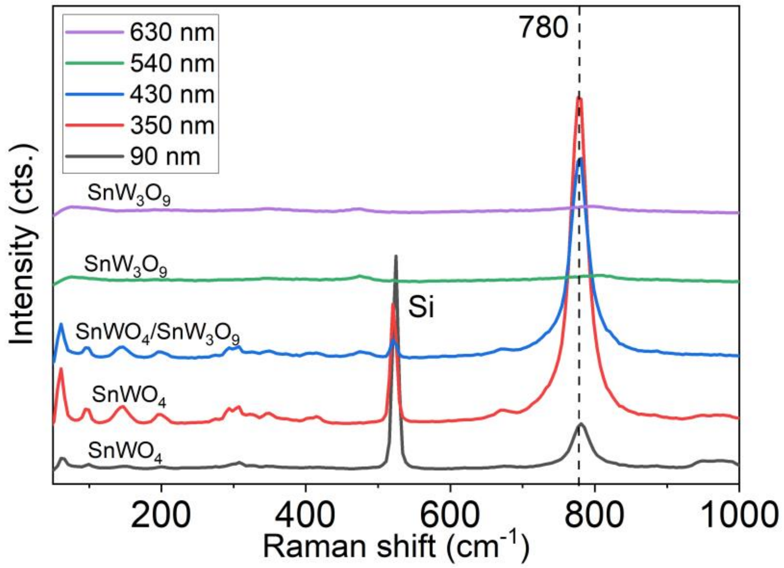

Figure S7. Raman spectra for different thick $SnWO_4$, $SnWO_4/SnW_3O_9$, and SnW_3O_9 films with different thicknesses prepared at 600 °C.

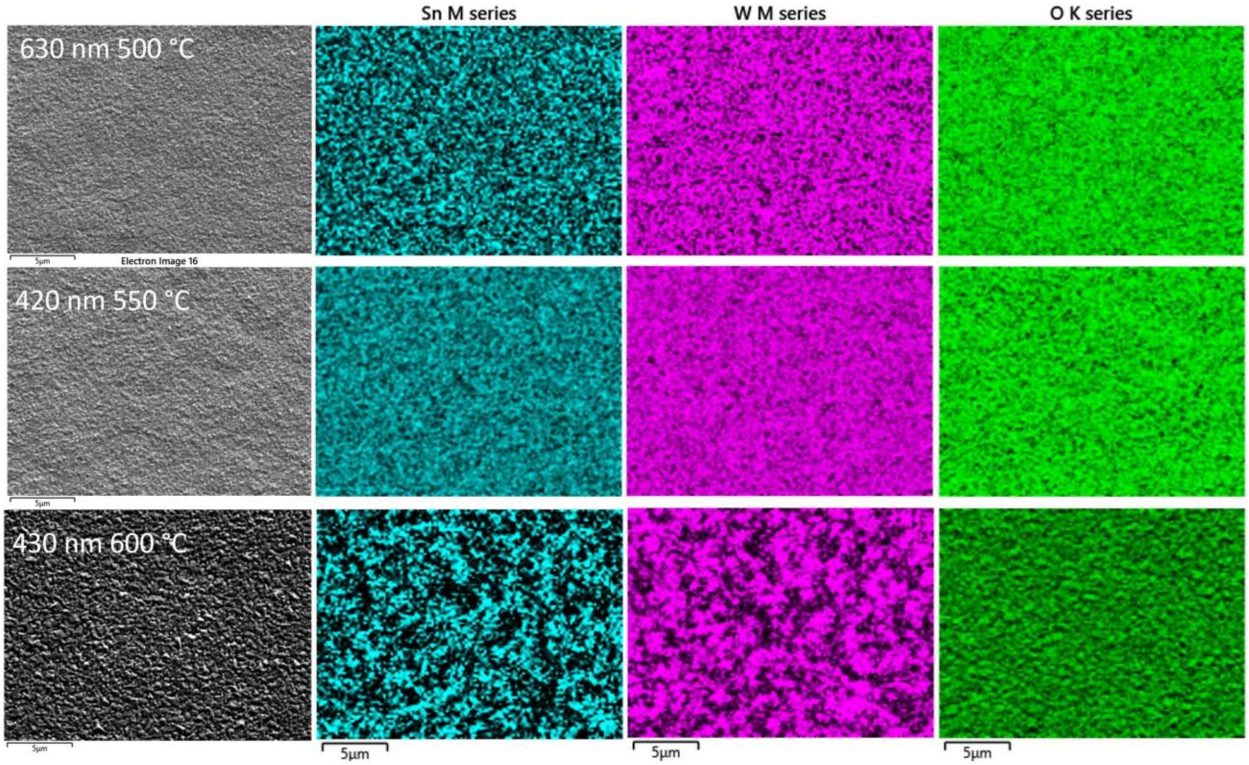

Figure S8. Elemental distribution of the films prepared at 500 °C, 550 °C, and 600 °C obtained by SEM and EDX mappings.

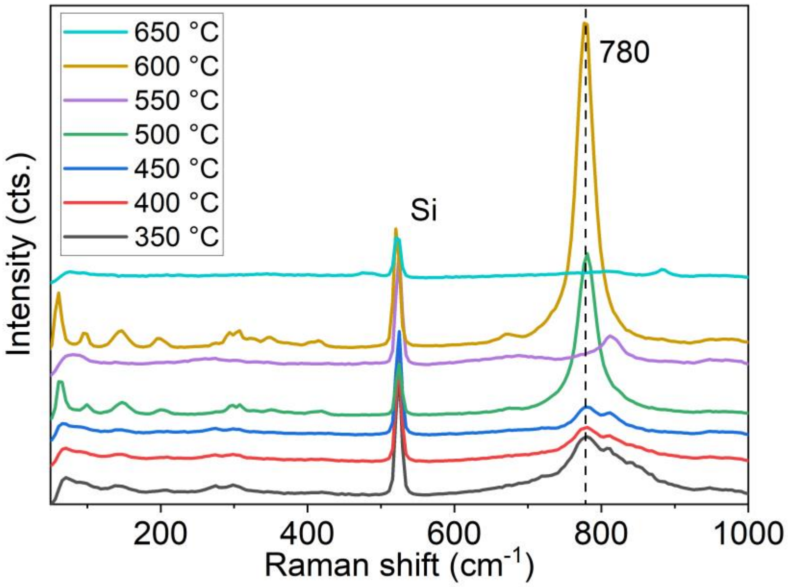

Figure S9. Raman spectra for the films prepared in the temperature range from 350 °C to 650 °C.

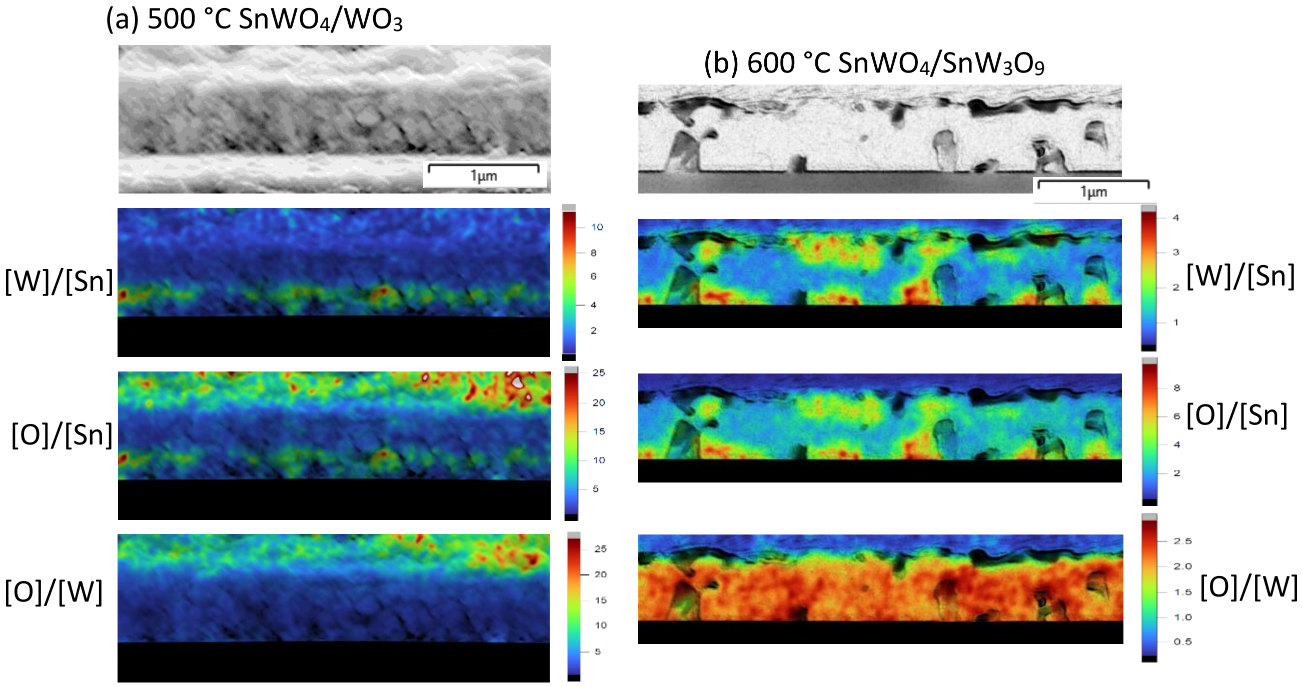

Figure S10. SEM and simulated elemental ratio from EDX images for (a) $SnWO_4/WO_3$, (b) $SnWO_4/SnW_3O_9$ films on n-Si prepared at 500 °C and 600 °C.

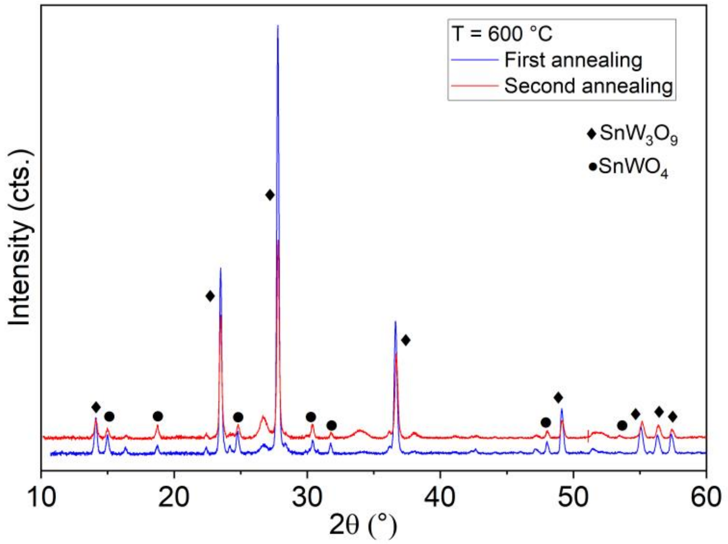

Figure S11. XRD patterns for $SnWO_4/SnW_3O_9$ films prepared on n-Si substrate at 600 °C.

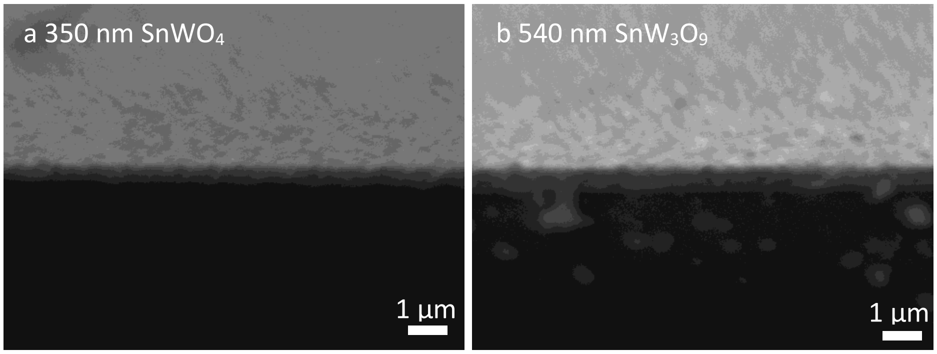

Figure S12. SEM cross section images for (a) 350 nm $SnWO_4$ and (b) 540 nm SnW_3O_9 films.

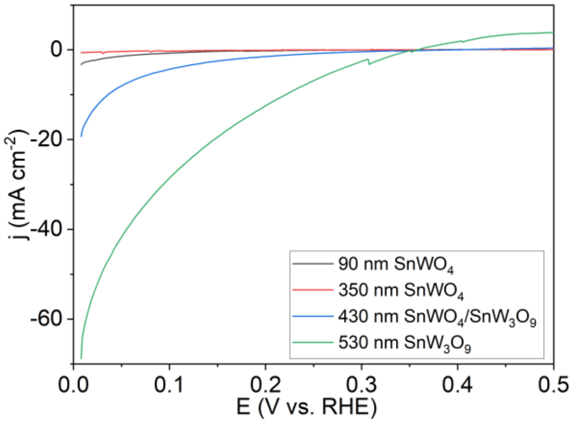

Figure S13. Linear scan voltammetry for (a) 90 nm $SnWO_4$, (b) 350 nm $SnWO_4$, (c) 435 nm $SnWO_4/SnW_3O_9$ and (d) 540 nm SnW_3O_9 thick films on n-Si in 0.5 M Na_2SO_4.

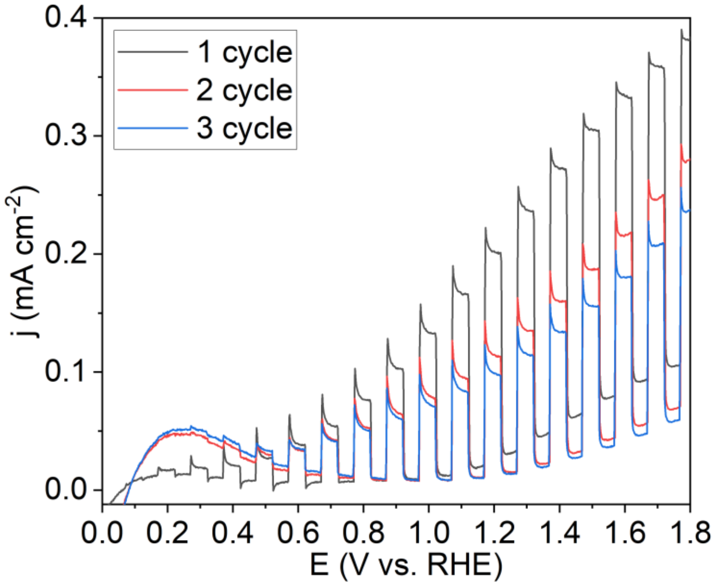

Figure S14. Linear scan voltammetry curves for a 520 nm $SnWO_4$ film prepared at 450 °C with on FTO substrates in 0.5 M $NaSO_4$ in 3 cycles.

Atomic layer deposition of TiO_2 thin films

Thermal atomic layer deposition was performed at 300 °C using a R-200 Advanced ALD system from Picosun with titanium tetrachloride (TiCl4, 22 °C, Dockweiler Chemicals electronic-grade) and water (22 °C) as precursors. The TiO2 thin films of 20 nm were obtained with 400 ALD cycles consisting of a dosing step with the Ti-containing precursor (0.1 s), a purge step with nitrogen (4.9 s, 300 sccm, 99.9999 %, Linde), a dosing step with water (0.1 s) and a final nitrogen purge step (13.4 s).

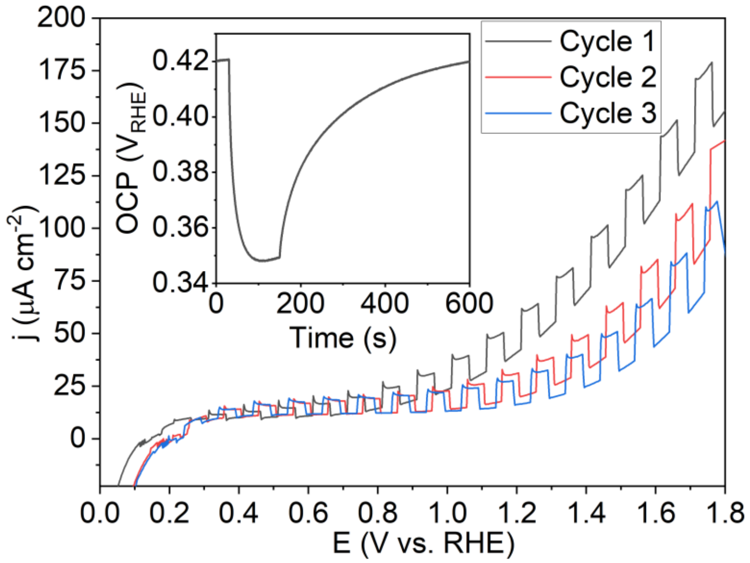

Figure S15. LSV and OCP curves for a 520 nm $SnWO_4/TiO_2$ film on FTO in 0.5 M Na_2SO_4.

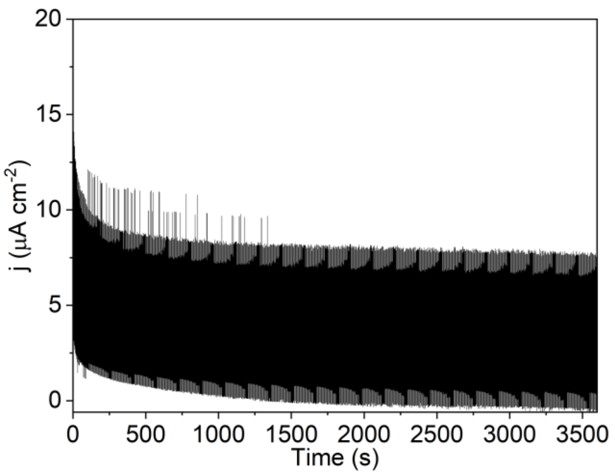

Figure S16. j as a function of time at 1.23 V_{RHE} for a 520 nm $SnWO_4/TiO_2$ film on FTO in 0.5 M Na_2SO_4. The light on and off times are 2 sec.

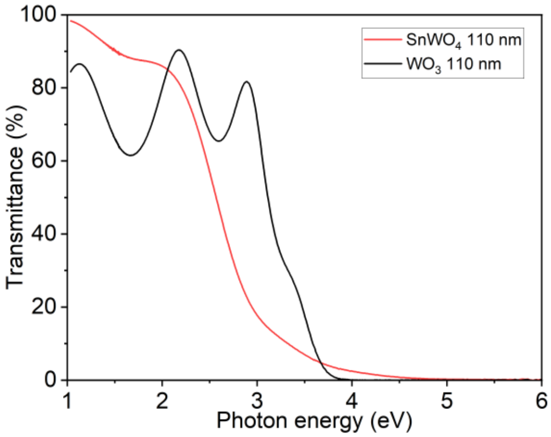

Figure S17. Transmittance spectra for both 110 nm thick WO_3 and $SnWO_4$ films on quartz.

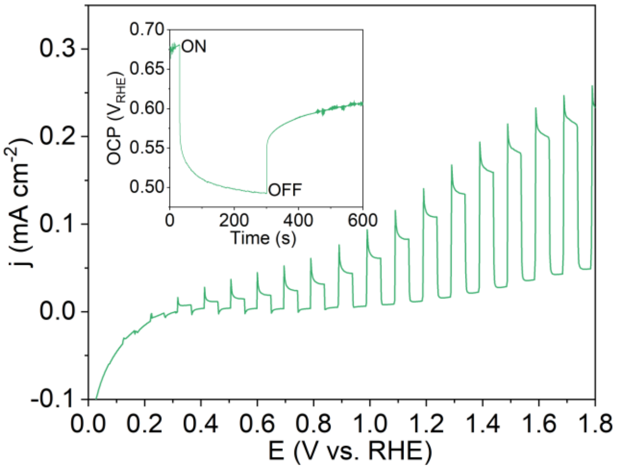

0.19 V

Figure S18. LSV and OCP curves for a 500 nm $SnWO_4$ film on TiN:O in 0.5 M Na_2SO_4.

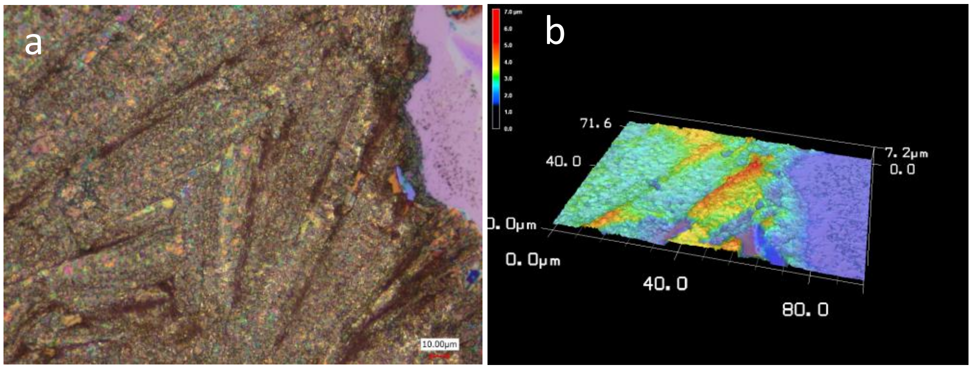

Figure 19. An optical image (a) and a topography (b) of the 500 nm thick $SnWO_4$ film on the TiN:O substrate prepared at 450 °C. The violet region shows the TiN:O substrate. The pristine thickness of W layer is 150 nm.

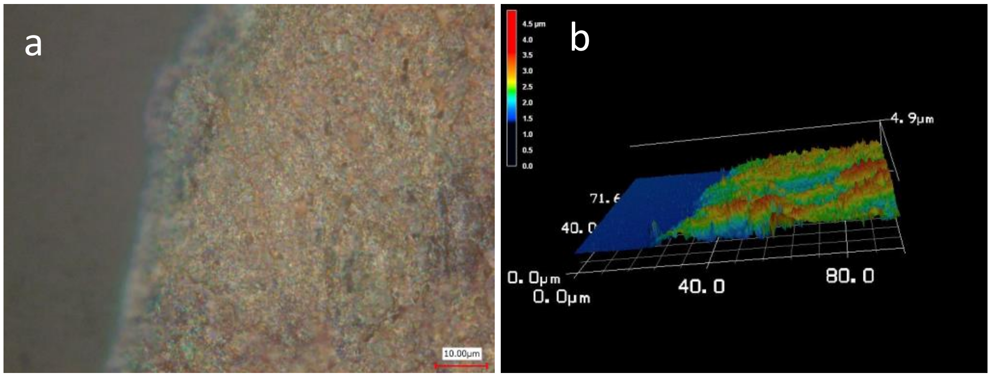

Figure S20. An optical image (a) and a topography (b) of the 520 nm thick $SnWO_4$ film on the FTO substrate. The blue region shows the FTO substrate prepared at 450 °C. The pristine thickness of W layer is 150 nm.

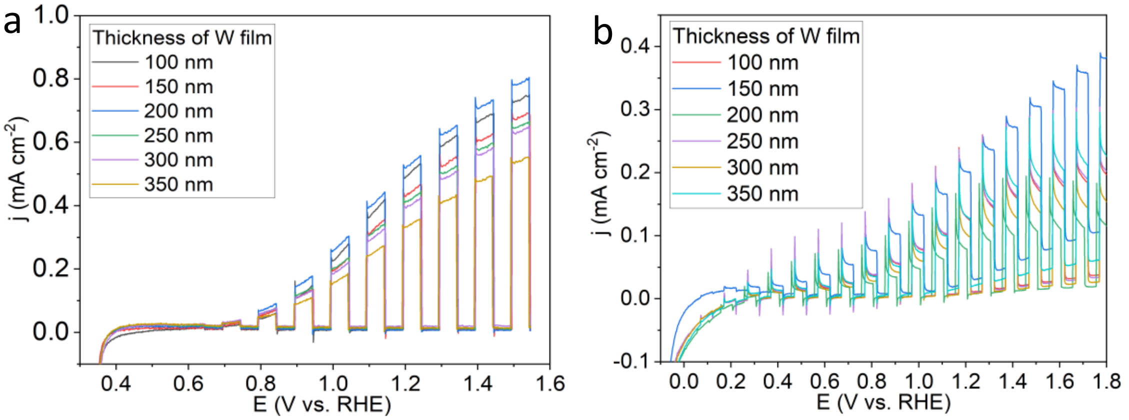

Figure S21. Linear scan voltammetry for different thick (a) WO_3 films prepared at 500 °C in air and transformed into (b) $SnWO_4$ films at 450 °C with $SnCl_2$ powder in vacuum on FTO substrates. The thicknesses are shown for pristine metal W films before oxidation and stannation. The $SnWO_4$ films have shown rough surfaces and therefore, it was difficult to evaluate the thickness of the films.

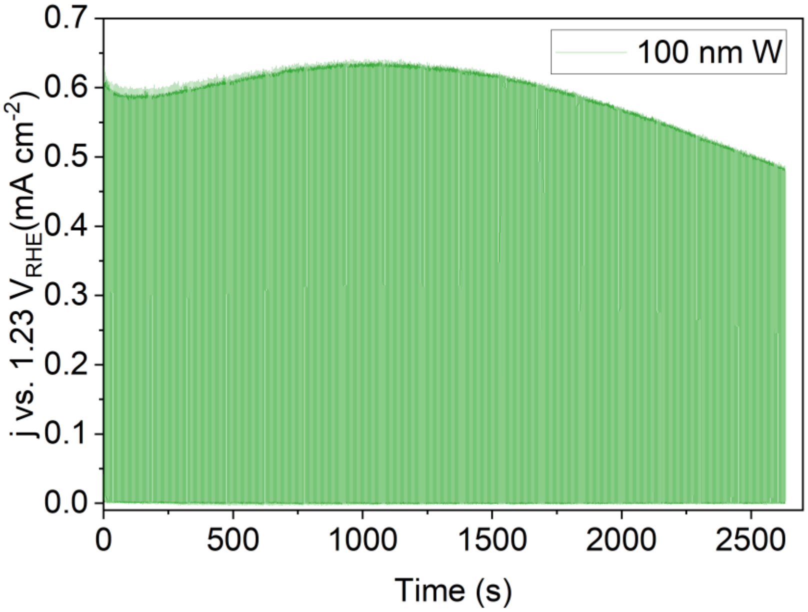

Figure S22. j as a function of time at 1.23 V_{RHE} for a 234 nm thick WO_3 film prepared at 500 °C in air on FTO.

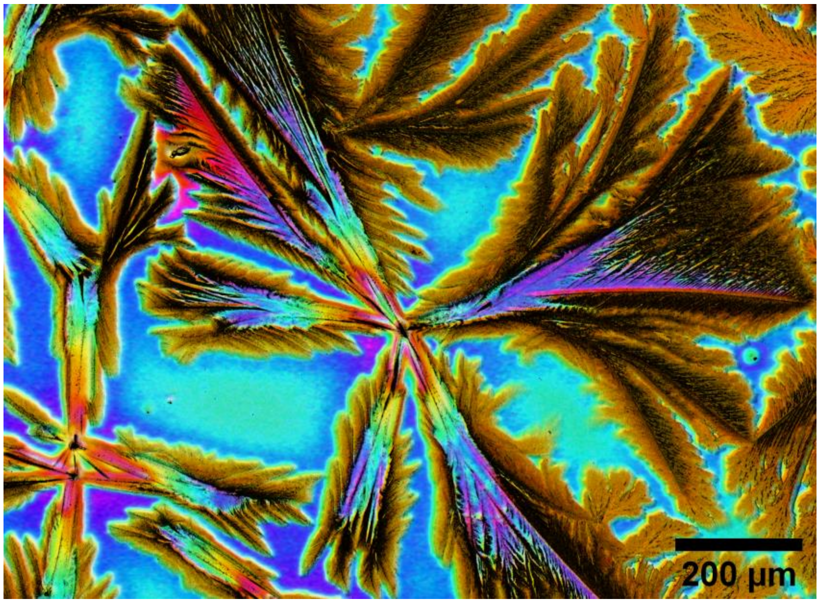

Figure S23. An optical image of the 100 nm thick $SnWO_4$ film on the quartz glass substrate prepared at 450 °C. The pristine thickness of W layer is 25 nm.

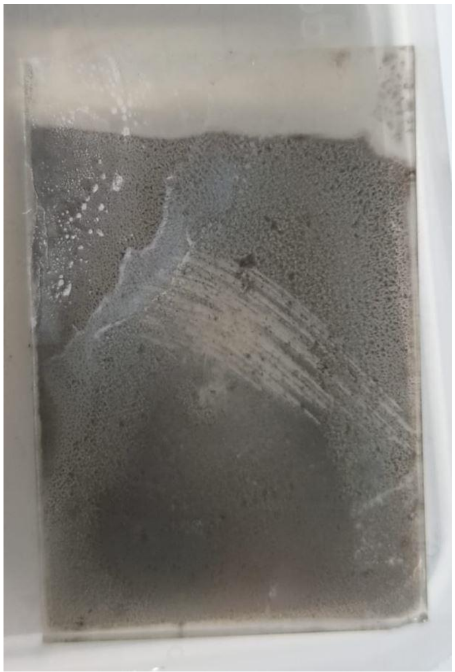

Figure S24. An image of the tin tungstate film prepared at 600 °C on FTO substrate. The film was scratched to find its weak adhesion.
